# The Arabidopsis KH-Domain RNA-Binding Protein ESR1 Functions in Components of Jasmonate Signalling, Unlinking Growth Restraint and Resistance to Stress

**DOI:** 10.1371/journal.pone.0126978

**Published:** 2015-05-18

**Authors:** Louise F. Thatcher, Lars G. Kamphuis, James K. Hane, Luis Oñate-Sánchez, Karam B. Singh

**Affiliations:** 1 CSIRO Agriculture Flagship, Centre for Environment and Life Sciences, Wembley, Western Australia, Australia; 2 The Institute of Agriculture, The University of Western Australia, Crawley, Western Australia, Australia; Oklahoma State University, UNITED STATES

## Abstract

Glutathione S-transferases (GSTs) play important roles in the protection of cells against toxins and oxidative damage where one Arabidopsis member, *GSTF8*, has become a commonly used marker gene for early stress and defense responses. A *GSTF8* promoter fragment fused to the *luciferase* reporter gene was used in a forward genetic screen for Arabidopsis mutants with up-regulated *GSTF8* promoter activity. This identified the *esr1-1* (*enhanced stress response 1*) mutant which also conferred increased resistance to the fungal pathogen *Fusarium oxysporum*. Through positional cloning, the *ESR1* gene was found to encode a KH-domain containing RNA-binding protein (At5g53060). Whole transcriptome sequencing of *esr1-1* identified altered expression of genes involved in responses to biotic and abiotic stimuli, hormone signaling pathways and developmental processes. In particular was an overall significant enrichment for jasmonic acid (JA) mediated processes in the *esr1-1* down-regulated dataset. A subset of these genes were tested for MeJA inducibility and we found the expression of some but not all were reduced in *esr1-1*. The *esr1-1* mutant was not impaired in other aspects of JA-signalling such as JA- sensitivity or development, suggesting ESR1 functions in specific components of the JA-signaling pathway. Examination of salicylic acid (SA) regulated marker genes in *esr1-1* showed no increase in basal or SA induced expression suggesting repression of JA-regulated genes is not due to antagonistic SA-JA crosstalk. These results define new roles for KH-domain containing proteins with ESR1 unlinking JA-mediated growth and defense responses.

## Introduction

Plants are subject to constant changes in their environment and rapid molecular responses to these are necessary for plant survival. Upon detection of abiotic or biotic stress a series of induced signalling pathways are activated, mediated by key signalling hormones such as salicylic acid (SA), jasmonic acid (JA) and abscisic acid (ABA), culminating in the expression of plant protectant and defense genes (reviewed by [1–8]). However, as multiple abiotic and biotic stresses can take place at the same time, a complex interplay of signalling pathways and responses can manifest resulting in opposing reactions. One mechanism to rapidly modify opposing stress-induced transcriptomes is to control the stability, degradation or turnover of specific transcripts at a post-transcriptional level through RNA-binding proteins.

RNA-binding proteins are mostly characterised by the presence of one or more RNA-binding domains. In addition to mRNA stability and decay, RNA-binding proteins are involved in diverse post-transcriptional processes including the maturation of mRNA through splicing, capping, polyadenylation and export from the nucleus [9, 10]. Along with plant specific processes such as flowering, the sessile nature of plants and a necessity to adapt quickly to changing environmental conditions may be why plants encode many RNA-binding, with over 200 RNA-binding proteins predicted in Arabidopsis [11]. Interestingly though, very few RNA-binding proteins have been functionally characterised in plants.

One group of genes expressed in response to biotic and abiotic stress are those belonging to the ubiquitous *GLUTATHIONE S-TRANSFERASE* (*GST*) enzyme family [12–15]. Plant GSTs protect tissues against oxidative damage or from toxic products typically by catalyzing the conjugation of glutathione to a variety of electrophilic substrates of endogenous or exogenous origin, rendering the substrate less toxic (reviewed by [13, 15]). Expression of the Arabidopsis *GSTPHI8* (*GSTF8*) gene is induced rapidly by diverse biotic and abiotic elicitors including pathogen attack, phytohormones, herbicides, heat and high-light stress, and as such has become a marker gene for early stress and defense responses [12, 16–23].

Using the *GSTF8* promoter to control the expression of a *Firefly Luciferase* reporter gene (*GSTF8*:*LUC*), we have been able to non-invasively monitor the plant’s stress status, primarily within root tissues where *GSTF8* is predominantly expressed [17, 19, 21, 24]. To identify mechanisms controlling *GSTF8*:*LUC* activity we conducted a forward genetic screen using mutagenized populations of plants containing *GSTF8*:*LUC* [23]. One mutant isolated from this screen, designated *disrupted in stress responses* (*dsr1*), exhibited loss of SA inducible *GSTF8*:*LUC* activity and increased susceptibility to several fungal and bacterial pathogens [23]. The *dsr1* mutation was mapped to a single amino acid change in a subunit of the mitochondrial energy machinery (complex II subunit SDH1-1), causing a reduction in induced reactive oxygen species production (ROS) from mitochondria and identifying mitochondrial derived ROS as a critical component of plant defense [23].

To complement the *dsr1* study, we screened for mutants with enhanced *GSTF8*:*LUC* expression in the aim of identifying mutants with increased tolerance to biotic stress. We identified several alleles of a mutant called *enhanced stress response 1* (*esr1*) encoding a K homology (KH) domain containing RNA-binding protein (At5g53060). The *esr1* mutants confer constitutive *GSTF8*:*LUC* expression and increased resistance to the root-infecting fungal pathogen *Fusarium oxysporum*. Detailed analysis of the *esr1-1* allele also identified significant down-regulation of genes enriched for involvement in JA-mediated responses. Other mutants of *At5g53060* are reported to confer altered tolerance to abiotic stress [25–27] such as heat stress which we also established for *esr1-1*. While many Arabidopsis mutants conferring increased resistance to specific pathogens have been identified, these are commonly associated with a consequential decrease in tolerance to abiotic stress, or fitness costs such as poor growth or yield [2, 6, 28, 29]. By contrast, *esr1-1* displays increased *F*. *oxysporum* resistance, heat tolerance, and lacked observable defects in growth or development. These results define new roles for ESR1/At5g53060, functioning in biotic stress responses, JA-signalling, and unlinking growth restraint and resistance to stress.

## Materials and Methods

### Plant material and growth conditions

Unless otherwise specified, all experiments were conducted with the *Arabidopsis thaliana* Columbia-0 transgenic line (JC66/*GSTF8*:*LUC*) containing 791 bp of the *GSTF8* promoter fused to a luciferase reporter [17, 24]. Seeds were surface-sterilized, stratified at 4°C, and plated onto 100-mm square agar plates containing Murashige and Skoog (MS) salts as described previously [17]. Plates for luciferase assays were supplemented with 50 uM luciferin (Biosynth AG). Plate and soil grown plants were incubated under a 16-h light/8-h dark cycle at 22°C. The T-DNA insertion mutant [30] used to generate *esr1-2* (SALK_095666) was obtained from the Arabidopsis Biological Resource Centre (ABRC). For generation of plants expressing candidate *ESR1* genes the *At5g53060*, *At5g53150* and *At5g52860* CDS were amplified using primers listed in S1 Table. The resulting amplicons were cloned into BamHI/EcoRI digested binary vector pKEN [31] and confirmed by sequencing. *35S*:*At5g53060* pKEN, *35s*:*At5g53150* pKEN and *35S*:*At5g52860* pKEN were mobilized into *Agrobacterium tumefaciens* AGL1 and transformed into *esr1-1* as per [31]. Transgenic plants were selected based on resistance to 10 ug/mL glufosinate ammonium (Fluka). To generate *esr1-2*, SALK_095666 and wild-type *GSTF8*:*LUC* lines were crossed and F_3_ seedlings homozygous for the T-DNA and *GSTF8*:*LUC* selected.

### Bioluminescence and luciferase assays

Seedling bioluminescence was captured and quantified by imaging in an EG & G Berthold molecular light imager as previously described [17, 21]. Biochemical luciferase assays were performed as described by [24]. For 1 mM SA (Sigma) or temperature (45°C) treatments, 7-day old seedlings grown on square 100-mm Petri dishes were either covered with the liquid treatment at room temperature or incubated in temperature controlled cabinets for 40 min. After this time, excess liquid was discarded from relevant plates and the plates imaged such that, after acquiring the 0 hour bioluminescence image, 1 hour had elapsed.

### Mutant screen and mapping of *enhanced stress response* 1

Mutagenesis of wild-type *GSTF8*:*LUC* was described by [23]. For mapping, a genetic cross between *esr1-1* and Ler was generated and initial mapping conducted on 35 homozygous *esr1-1* F_2_ plants (exhibiting constitutive *GSTF8*:*LUC* activity) with a set of 18 simple sequence-length polymorphism (SSLP) markers to map *esr1-1* to the bottom of chromosome 5, linked to marker *ciw9*. Additional mapping was performed by screening 1040 homozygous F_2_ plants with markers listed in S1 Table.

### DNA isolation, Illumina sequencing, assembly and SNP annotation

DNA was extracted from backcrossed *esr1-1* using the CTAB method as described previously [32], followed by purifications using Agencourt AMPure XP beads (Beckman Coulter). Illumina Truseq DNA libraries were generated using manufactures recommendations and sequenced on an Illumina HiSeq1000 platform. Reads were trimmed, mapped against the TAIR10 release of the Arabidopsis genome [33] using bowtie2 v2.0.0b7 (parameters:-sensitive—end-to-end—met-stderr) [34] and SAMtools [35]. The aligned sequences were scanned for SNPs relative to the TAIR10 reference using GATK (v2.1-6-g6a46042) [36]. The potential for SNP errors occurring around insertion-deletion regions was reduced using GATK RealignerTargetCreator (parameters: --windowSize 20 --minReadsAtLocus 2) and IndelRealigner (parameters: consensusDeterminationModel USE_SW –LODThresholdForCleaning 2 –maxconsensuses 100 –maxReadsForRealignment 100000 –maxReadsInMemory 300000). Alignments were searched for SNPs using UnifiedGenotyper (parameters:—stand_call_conf 50.0 –stand_emit_conf 10.0). SNPs were considered as potentially contributing to the *esr1-1* phenotype if they resided within the *esr1-1* mapped loci. For *esr1-3* and *esr1-4*, pooled DNA from 50–60 homozygous F_2_ plants from a Ler outcross were sequenced at 60–70x coverage by the Australian Genome Research Facility (AGRF) using an Illumina HiSeq Platform. Between 77.9 and 80.2 million paired-end reads (100 bp in length) per sample were mapped to the Arabidopsis TAIR10 genome reference sequence, SNPs called using the recommended SAMtools mpileup script and processed through the NGM tool http://bar.utoronto.ca/ngm/ [37].

### Developmental and MeJA root elongation inhibition assays

Seeds of wild-type *GSTF8*:*LUC*, *esr1-1* and *esr1-2* were surface sterilized and plated onto MS media with germination rates measured as a percentage of total seeds plated (n = 60–70). For root length and MeJA root elongation inhibition assays, seeds were sterilized as above and plated onto MS media in either the presence or absence of 25 or 50 μM MeJA. Root length was measured on 7-day old seedlings using ImageJ [38]. Flowering time assays were conducted under long day conditions16-h light/8-h dark cycle at 22°C (n = 10).

### Pathogen assays

For *F*. *oxysporum* inoculations the isolate Fo5176 was used. Root-dip inoculations on 4-week-old plants with a 1x10^6^ cell/mL spore suspension were performed as described previously [39–41]. *A*. *brassicicola* assays were performed with isolate UQ4273 as described by [23]. A 5 ul portion of a 1x10^6^ cell/mL spore suspension was applied to leaves of 3- to 4-week-old plants. Mock treatments with potato dextrose broth (PDB) were also conducted. Lesion size was measured with ImageJ [38]. For *R*. *solani* inoculations the strains AG2 or AG8 were used as previously described [23, 42]. 7-day old seedlings were sown into vermiculite and inoculated with 1 mL of 1x10^6^ cell/mL mycelium suspension.

### RNA isolation

For qRT-PCR and RNAseq experiments on untreated plants, tissue was collected from 4-, 7- or 14-day old seedlings grown vertically on MS agar plates. For gene expression under MeJA or SA treatment, 12-d-old seedlings germinated on MS plates were gently lifted into a mock medium (MS broth), 100 uM MeJA medium (MS broth plus MeJA), or 1 mM SA (MS broth plus SA) such that the roots were submerged, and left for 6 or 24 h before harvesting. Three separate biological replicates were taken for all experiments consisting of whole seedlings pooled from 20–30 seedlings grown at the same time in the same environment, then frozen in liquid nitrogen, and stored at 80°C. RNA isolation was performed using the Qiagen RNeasy Plant Mini Kit (Qiagen). DNase treatment was performed after RNA isolation using TURBO DNase followed by treatment with the DNase Inactivation reagent (Ambion).

### qRT-PCR

Following RNA isolation and DNase treatment, complementary DNA synthesis was performed using SuperscriptIII reverse transcriptase (Invitrogen) with oligo(dT) (Invitrogen) and RNasin (Promega) with 1ug of input RNA. qRT-PCR was performed using SsoFast EvaGreen Supermix (Bio-Rad) on a CFX384 (Bio-Rad) system. Thermoycling and melt-curve conditions are described by [43]. Absolute gene expression levels relative to the previously validated [41, 44, 45] reference gene mix *β-actin2*, *β-actin7*, and *β-actin8* (*At1g49240*, *At3g18780*, and *At5g09810*, respectively) were used for each complementary DNA sample using the equation: relative ratio gene of interest/actin = (Egene^-Ct gene^)/(Eactin^-Ct actin^) where Ct is the cycle threshold value. The *β-actin* mix contains reverse primers for each of the three *β-actin* genes and a universal forward primer. The mean expression range of the reference gene was found to be within ±1 Ct across all samples. Several gene-specific primer sequences are previously published [45, 46] and are also listed in S1 Table.

### RNA-seq library construction, Illuminia sequencing and identification of differentially expressed genes

Following RNA isolation and DNase treatment of 14-day old wild-type or *esr1-1* samples, Illumina TruSeq libraries were generated from 1 μg of total RNA and sequenced on a HiSeq1000 platform (Illumina). RNA-seq paired-end reads were trimmed for low-quality base-calls and Illumina adapter sequences via Cutadapt (v1.1, parameters: --quality-cutoff 30 --overlap 10 --times 3 –minimum-length 25) [47]. Reads trimmed to less than 25 bp were discarded and remaining reads sorted into pairs and singleton reads. RNA-seq reads were mapped to the TAIR10 Arabidopsis genome reference [33] via Tophat (v2.0.9, parameters: --b2-very-sensitive-r 50 --mate-std-dev 100-i 20-I 4000-g 20 --report-secondary-alignments --report-discordant-pair-alignments-m 0 --min-coverage-intron 20 --coverage-search --microexon-search --library-type fr-unstranded) [48]. RNAseq read alignments were supplied to Cuffdiff (Cufflinks v2.1.1) [49] to calculate normalised expression within the TAIR10 annotated genes as fragments per kilobase of transcript per million mapped fragments (FPKM) (parameters: --frag-bias-correct --min-frags-per-transfrag 4 --multi-read-correct). Significantly differentially expressed transcripts between wild-type and *esr1-1* were detected from 3 biological replicates using Cuffdiff with default Benjamini-Hochberg correction for multiple-testing, based on a False Discovery Rate ≤0.05. Functional annotations of genes and AGI symbols were sourced from TAIR10 datasets. RNA-seq reads have been deposited in the NCBI Sequence Read Archive under BioProject ID SRP056904.

## Results

### Identification of the constitutive *GSTF8*:*LUC* mutant *esr1-1*

**Fig 1:**
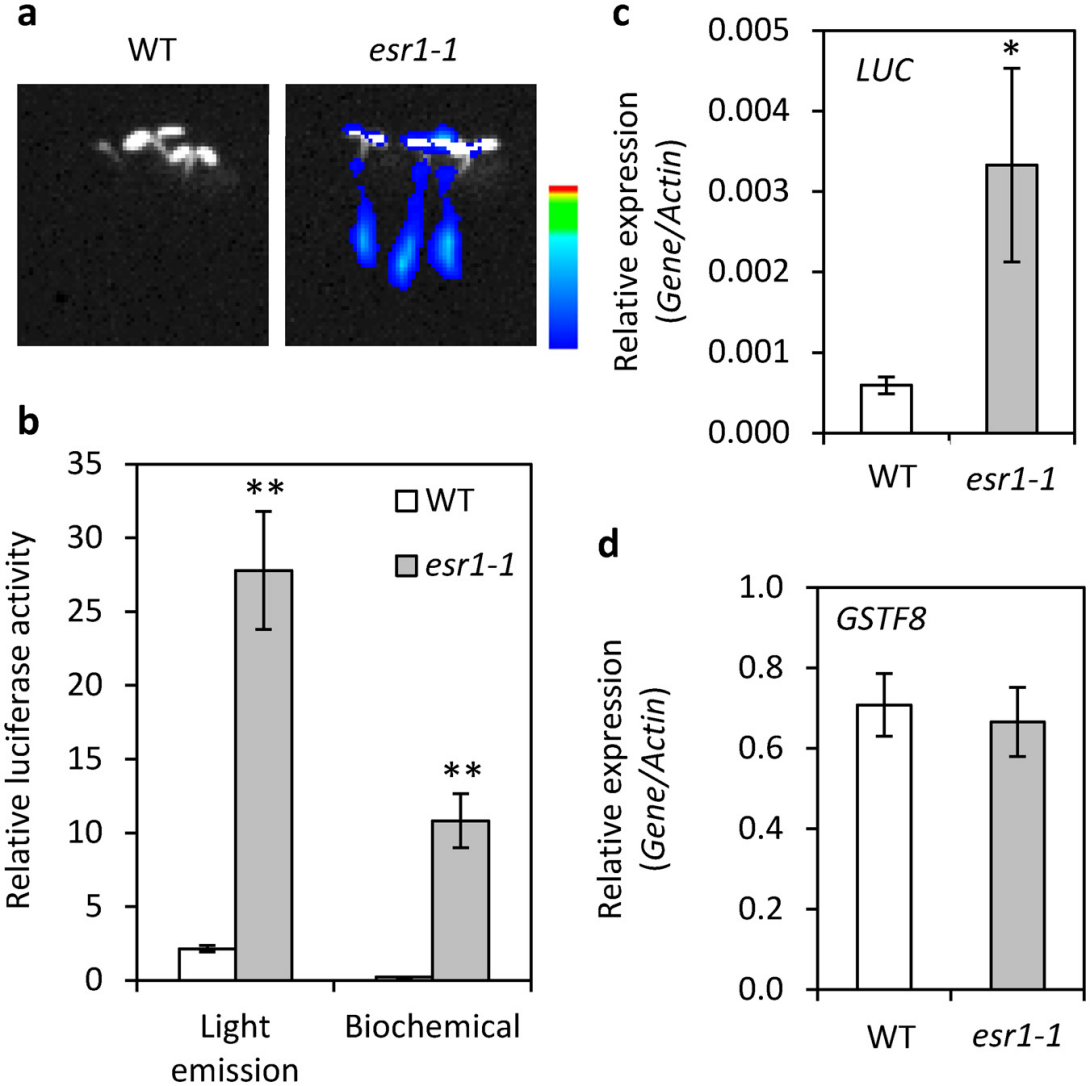
*esr1-1* causes hyper-expression of basal *GSTF8*:*LUC* activity. (a) *GSTF8*:*LUC* expression in 4 day old wild-type (WT) and *esr1-1* seedlings. Shown is bioluminescence (pseudocolored blue) superimposed onto a fluorescence (white) image. Intensity of bioluminescence ranges from blue to red as depicted in the intensity ruler. (b) Quantification of bioluminescence via *in vivo* light emission (relative light units/seedling; values are averages ± SE (n = 30) from 4 day old seedlings) and *in vitro* biochemical assays (units/20sec/mg protein; values are averages ± SE (n = 30) from 9 day old seedlings). (c-d) *Luciferase* (*LUC*) and *GSTF8* expression in 4 day old seedlings (values are averages ± SE of 4 biological replicates consisting of pools of 20 seedlings). Gene expression levels are relative to the internal control *β-actin* genes. Asterisks indicate values that are significantly different (**P<0.01, *P<0.05 Student’s *t-*test) from WT.

To identify negative regulators of root stress responses we screened mutants from an ethyl methansulfonate (EMS) mutagenised *GSTF8*:*LUC* population [23] for enhanced basal luciferase expression. Over 50 mutants with constitutive *GSTF8*:*LUC* expression were identified and termed *enhanced stress response* (*esr*) mutants. One of the mutants with the highest basal *GSTF8*:*LUC* expression (*esr1-1*) was further analysed and its phenotype confirmed in the M_3_ generation (Fig 1a and 1b). Quantitative real-time RT-PCR (qRT-PCR) was performed to confirm *LUCIFERASE* (*LUC*) gene expression and determine endogenous *GSTF8* expression. While *LUC* expression was up-regulated (5.6-fold greater than wild-type), *GSTF8* expression was unaltered (Fig 1c and 1d), suggesting the *esr1-1* mutation may only affect the *GSTF8*:*LUC* transgene and not endogenous *GSTF8* expression.

For cloning and heritability studies, we out-crossed *esr1-1* to the Landsberg *erecta* ecotype (Ler). All F_1_ plants showed the wild-type phenotype, and F_2_ plants displayed a ~3:1 segregation (59:21, χ^2^ test *p* = 0.8) suggesting the *esr1-1* phenotype is due to a recessive mutation in a single nuclear gene.

### *GSTF8*:*LUC* activity and endogenous *GSTF8* expression is up-regulated in *esr1-1* following SA treatment

**Fig 2:**
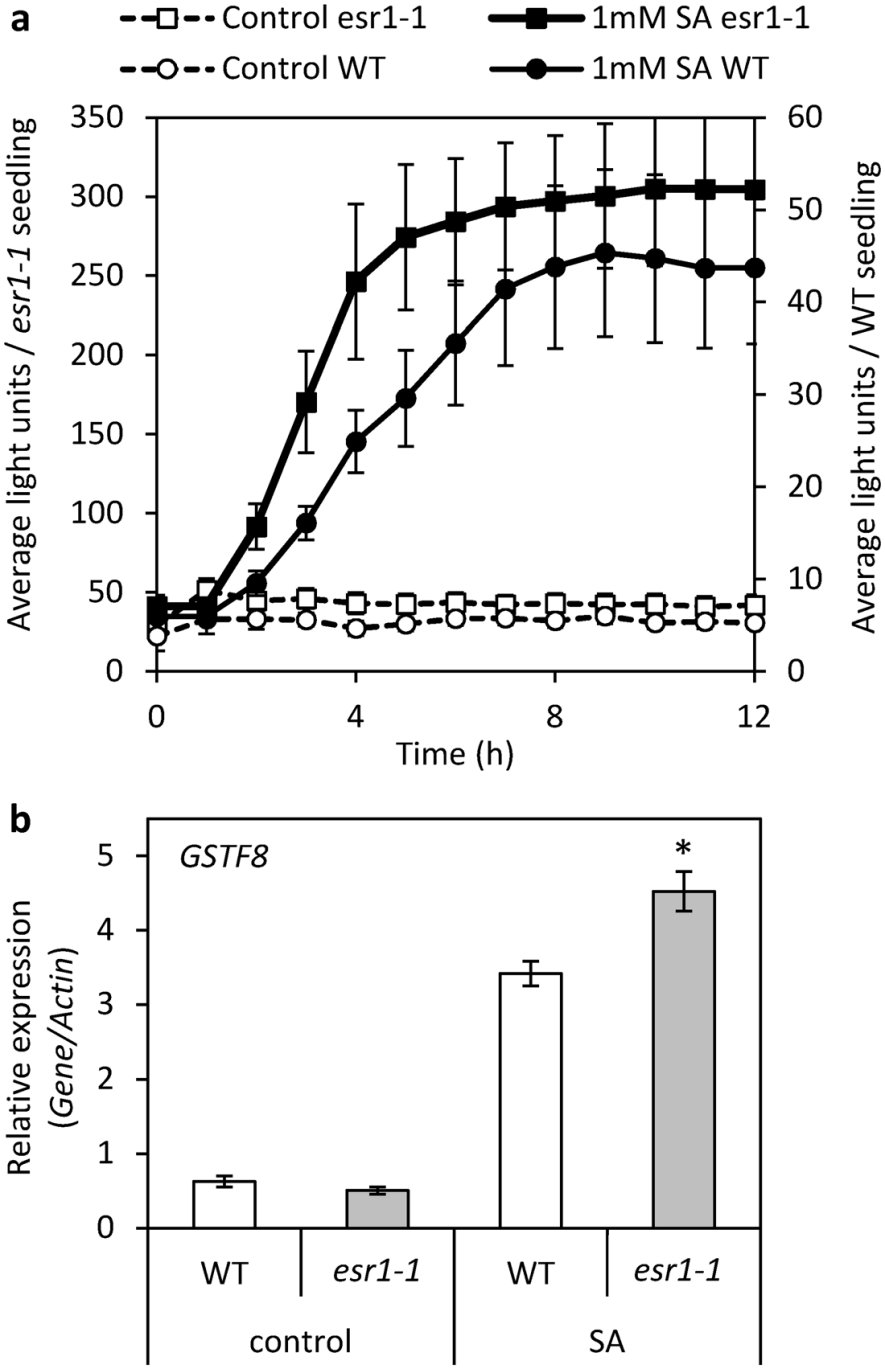
*GSTF8*:*LUC* activity and endogenous *GSTF8* expression is up-regulated in *esr1-1* following SA treatment. (a) Average *GSTF8*:*LUC* expression per wild-type (WT) and *esr1-1* seedling per hour after treatment with 1mM salicylic acid (SA) or a control treatment. Values are averages ± SE (n = 5) from 7 day old seedlings with *esr1-1* and WT values plotted on the left and right axes respectively. Similar results were obtained in independent experiments. (b) *GSTF8* expression in 12 day old seedlings 6 hours post control or SA treatment (values are averages ± SE of 3 biological replicates consisting of pools of 20–30 seedlings). Gene expression levels are relative to the internal control *β-actin* genes. Asterisks indicate values that are significantly different (*P<0.05 Student’s *t-*test) from WT.

To further characterise *esr1-1*, we monitored *GSTF8*:*LUC* expression following SA treatment, known to rapidly induce *GSTF8* promoter activity in wild-type plants [17, 24]. *GSTF8*:*LUC* activity increased more rapidly in *esr1-1* following SA treatment where it plateaued at 6–7 hours post treatment compared to wild-type seedlings where this occurred at 8–9 hours (Fig 2a). Expression of the endogenous *GSTF8* gene in *esr1-1* under SA-inducing conditions was also significantly higher in *esr1-1* compared to wild-type (Fig 2b). Combined with the lack of increased basal *GSTF8* expression in *esr1-1* (Fig 1d), these results suggest regulation of basal but not stress inducible *GSTF8 promoter*:*LUC* activity differs from the context of the endogenous *GSTF8* gene, possibly due to regulatory components beyond the promoter fragment used in this study.

### ESR1 is a negative regulator of resistance to the fungal pathogen *Fusarium oxysporum*

**Fig 3:**
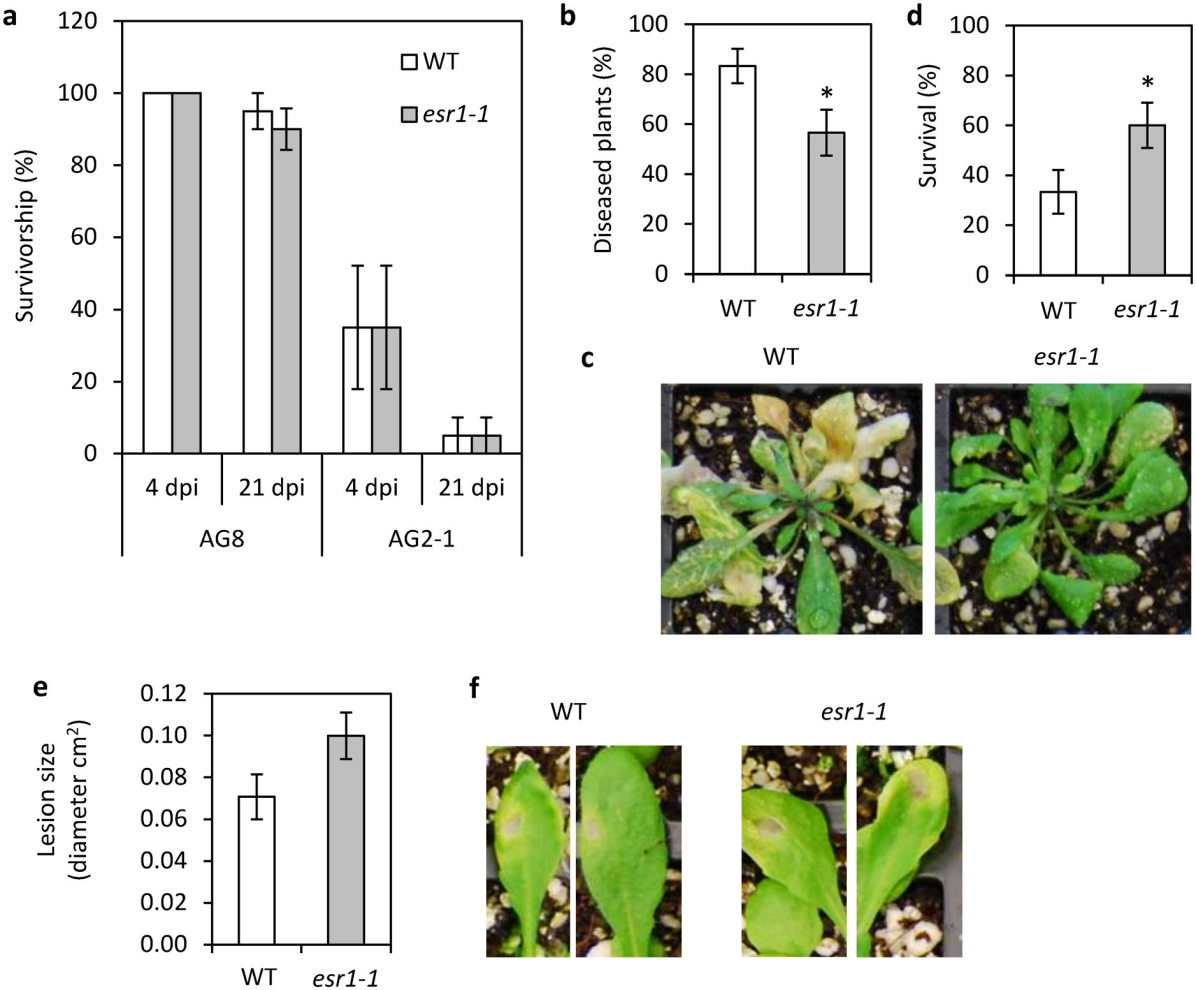
*esr1-1* exhibits increased resistance to *Fusarium oxysporum*. (a) Percentage survivorship of wild-type (WT) and *esr1-1* seedlings at 4 and 21 days post inoculation (dpi) with *Rhizoctonia solani* isolates AG8 and AG2-1. Values are averages ± SE of 4 biological replicates consisting of pools of 5 seedlings. (b-d) Disease phenotypes of *F*. *oxysporum* inoculated plants with (b) percentage and (c) representative images of diseased plants 10 days post inoculation. (d) Survival at 21 days post inoculation. Values are averages ± SE (n = 30). (e-f) *A*. *brassicicola* induced lesions on (e) WT and *esr1-1* leaves 3 days post inoculation with (f) representative images of leaves. Values are averages ± SE of 6 biological replicates consisting of lesion diameters measured from 4 inoculated leaves per plant. Asterisks indicate values that are significantly different (*P<0.05 Student’s *t-*test) from WT. Similar results were obtained in independent experiments.

We previously identified a mutant from the same screen as *esr1-1* but with loss of SA inducible *GSTF8*:*LUC* activity. This mutant termed *disrupted in stress response*s *1* (*dsr1*) exhibits increased susceptibility to several pathogens [23]. As *esr1-1* exhibits increased root localised *GSTF8*:*LUC* expression (Figs 1 and 2), we hypothesized *esr1-1* may confer increased resistance to root pathogens. To test this, we first inoculated wild-type and *esr1-1* plants with the root-infecting fungal pathogens *Rhizoctonia solani* and *Fusarium oxysporum* [19, 50]. While no significant difference in disease symptom development or survivorship was observed between wild-type or *esr1-1* plants inoculated with *R*. *solani* (strains AG2 or AG8) (Fig 3a), *esr1-1* did exhibit increased resistance to *F*. *oxysporum* (Fig 3b–3d). This was observed through both a delay in disease symptom development and increased survival. While jasmonate (JA)-mediated defences are required for resistance to most fungal necrotrophic pathogens (e.g. *Botrytis cinerea*, *Alternaria brassicicola*, [51]), JA-signalling confers susceptibility to *F*. *oxysporum* with mutants compromised in JA-dependant responses exhibiting resistance to this pathogen [41, 45, 52].

The increased resistance to *F*. *oxysporum* prompted us to determine if *esr1-1* conferred increased susceptibility to *A*. *brassicicola*. Larger *A*. *brassicicola* induced lesions were observed on *esr1-1* leaves compared to wild-type (Fig 3e and 3f), however not a statistically significant level. This phenotype was observed over several independent experiments suggesting ESR1 contributes a small affect to inhibition of *A*. *brassicicola* lesion development. As could be hypothesized from the *F*. *oxysporum* results, increased *A*. *brassicicola* induced lesions may be due to reduced JA-responses in *esr1-1*.

### *ESR1* encodes a K homology (KH) domain RNA-binding protein

**Fig 4:**
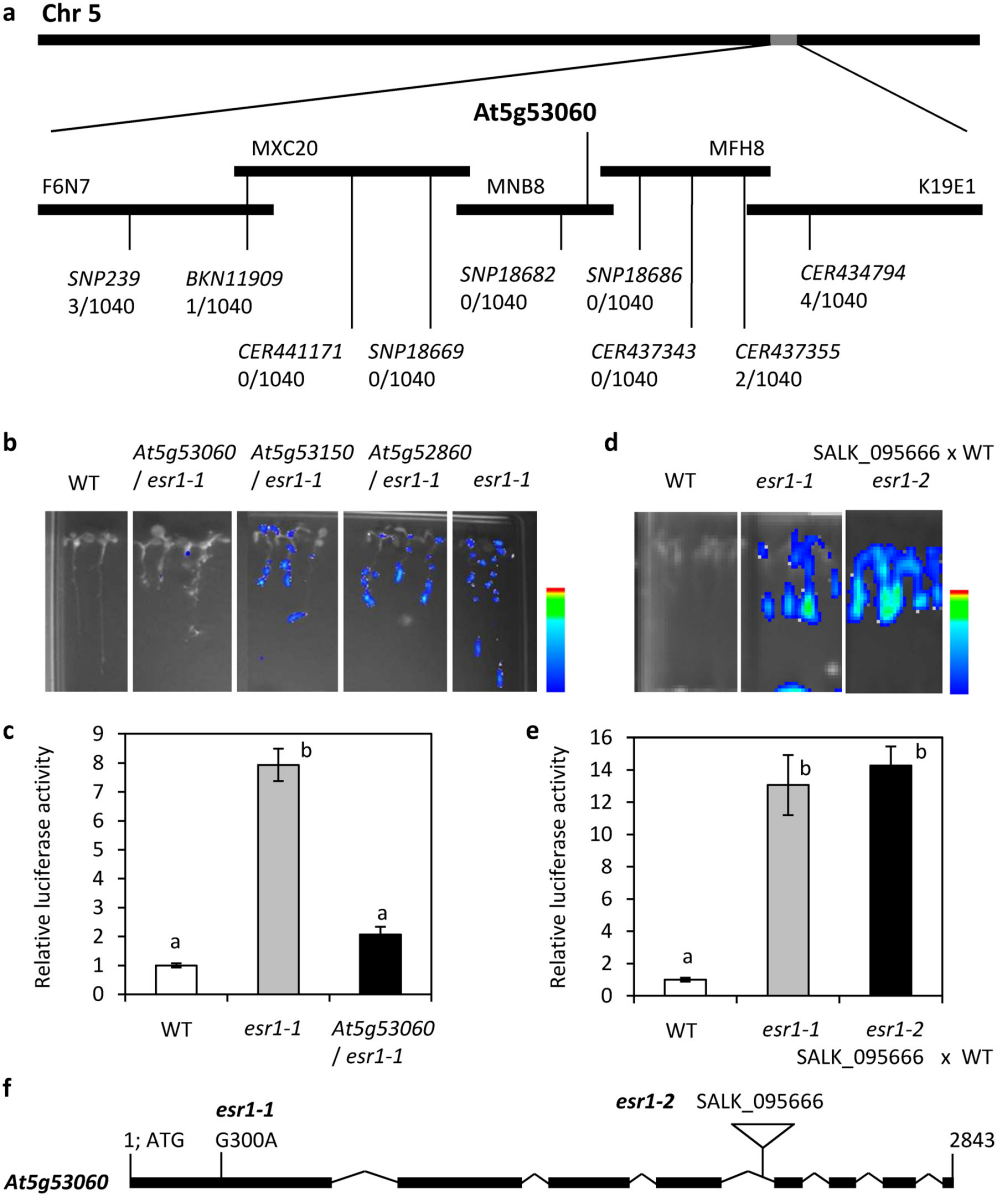
Molecular cloning of *esr1-1*. (a) Fine mapping of *esr1-1* with recombination events from 1040 plants analysed for each marker shown. (b-c) Wild-type (WT) and *esr1-1* genomes were sequenced and inspected for SNP differences within the mapped loci to identify 3 candidate genes. Molecular complementation of the *esr1-1* mutation by the wild-type *At5g53060* gene with (b) images and (c) luciferase quantification shown (P<0.05, all pairs Student’s t-test). (d-e) Genetic complementation between *esr1-1* and a *At5g53060* T-DNA knockout (SALK_095666) with (d) images and (e) luciferase quantification shown (*P*<0.05, all pairs Student’s t-test). The *esr1-2* mutant is an F_3_ line from a cross between WT plants and the T-DNA insertion line SALK_09566 and is homozygous for the T-DNA insertion and *GSTF8*:*LUC* transgene. (f) Structure of the *At5g53060* gene with *esr1-1* mutation and T-DNA knockout locations indicated. Filled boxes indicate exons, joining lines indicate introns. Positions are relative to the start codon.

To determine the causal *esr1-1* mutation, map based cloning was initiated using F_2_ seeds of the *esr1-1* and Ler cross. Genetic mapping was conducted on 1040 homozygous F2 plants. The mutation was narrowed down to a region on Chromosome 5 spanning ~200 Kb across three Bacterial Artificial Chromosomes (BACs); MXC20, MNB8 and MFH8 (Fig 4a). Whole genome sequencing of the *GSTF8*:*LUC* wild-type parent and *esr1-1* and alignment to the TAIR10 genome identified five single nucleotide polymorphisms (SNPs) within the mapped loci with three causing non-synonymous mutations in gene coding regions. Molecular complementation assays were conducted by individually introducing the three candidate genes under the control of the 35S promoter into the *esr1-1* background. The *35S*:*At5g53060* construct eliminated the *esr1-1* constitutive *GSTF8*:*LUC* expression and restored the wild-type phenotype, suggesting a G300A nucleotide change in *At5g53060* and resulting stop codon substitution of W100* causes the *esr1-1* phenotype (Fig 4b, 4c and 4f). *At5g53060* encodes a heterogenous nuclear riboprotein (hnRNP) K homology (KH) domain containing RNA-binding protein. The protein contains five KH-domains and the *esr1-1* mutation disrupts the first of these domains. KH-domains are found in many RNA-binding proteins and are associated with transcriptional and post-transcriptional processes where they can bind RNA or single stranded DNA [11, 53, 54]. In all subsequent experiments the *esr1-1* line used is a line backcrossed twice to the wild-type parent (to remove other EMS induced mutations) and has the same *GSTF8*:*LUC* phenotype as the M_3_ *esr1-1* line (data not shown).

**Fig 5:**
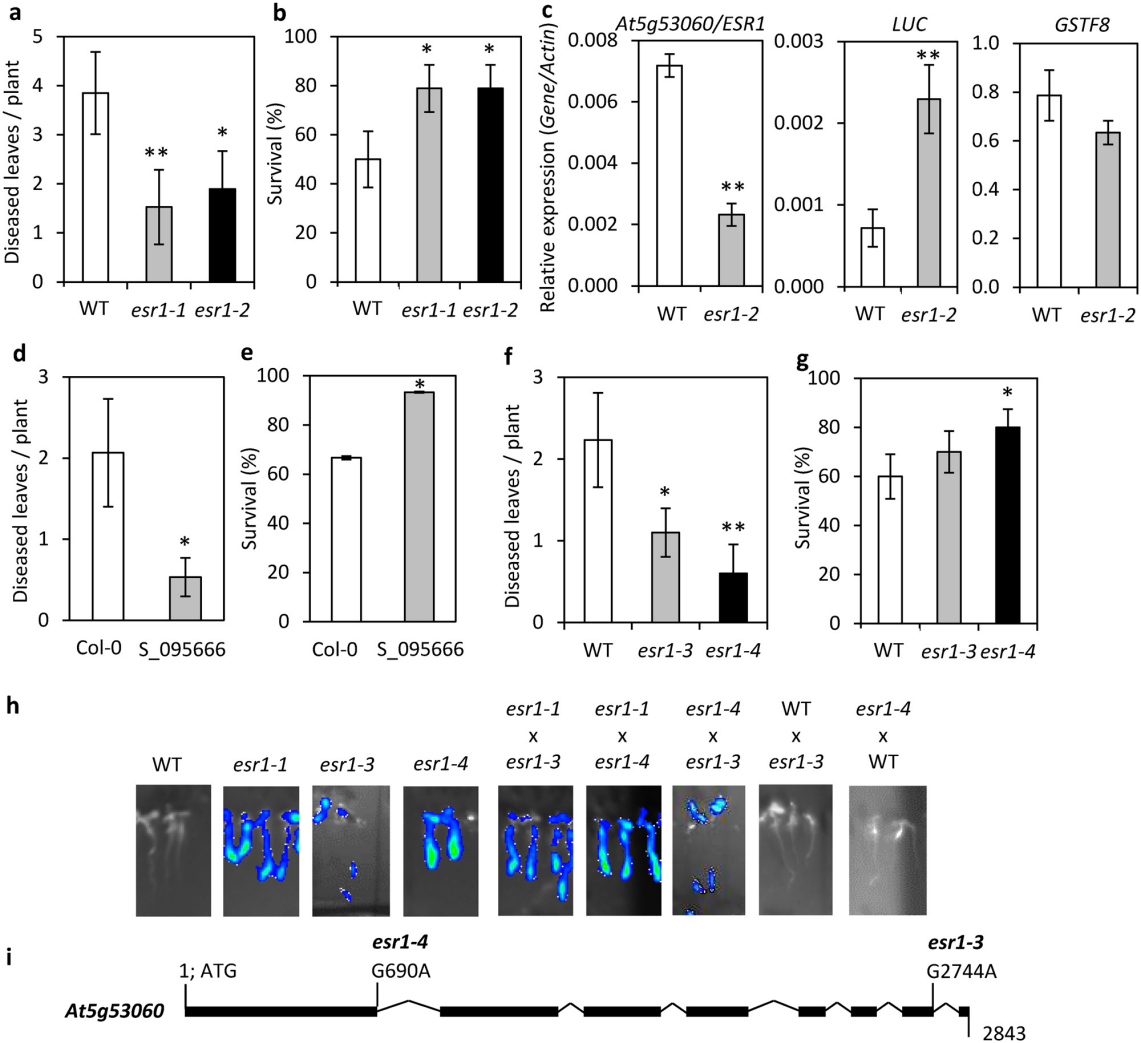
Identification and screening of other *esr1* alleles/insertion lines conferring increased resistance to *Fusarium oxysporum*. (a-b) Disease phenotypes of *F*. *oxysporum* inoculated wild-type (WT) *GSTF8*:*LUC* and *esr1-2* plants. Values are averages ± SE (n>15). (c) *At5g53060/ESR1*, *LUCIFERASE* (*LUC*) and *GSTF8* expression in 12 day old WT and *esr1-2* seedlings (values are averages ± SE of 3 biological replicates consisting of pools of 20 seedlings). Gene expression levels are relative to the internal control *β-actin* genes. (d-g) Disease phenotypes of *F*. *oxysporum* inoculated (d-e) Col-0 and SALK_09566, and (f-g) WT *GSTF8*:*LUC* and *esr1-3* and *esr1-4* plants. Values are averages ± SE (n>15). (h) *esr1-1*, *esr1-3* and *esr1-4* mutants were crossed and F_1_ progeny screened for complementation of the *GSTF8*:*LUC* constitutive expression phenotype. Crosses to wild-type (WT) *GSTF8*:*LUC* were included as controls. (i) Next Generation Mapping identified *esr1-3* and *esr1-4* mutations at splice site junctions in *At5g53060/ESR1*. For *Fusarium* disease assays, diseased leaves was measured at 14 days post inoculation and survival at 21 days post inoculation. Asterisks indicate values that are significantly different (**P<0.01, *P<0.05 Student’s *t-*test) from WT or Col-0.

To confirm a mutation in *At5g53060* is responsible for the *esr1-1* increased *GSTF8*:*LUC* and *Fusarium* resistance phenotypes, a T-DNA insertion line in *At5g53060* (SALK_095666) was crossed with wild-type *GSTF8*:*LUC*. We generated F_3_ seedlings homozygous for the T-DNA and *GSTF8*:*LUC* (subsequently named *esr1-2*) which displayed both the constitutive *esr1-1* luciferase phenotype and increased resistance to *F*. *oxysporum*, further supporting that the *esr1-1* mutant phenotypes result from disruption of *At5g53060* (Figs 4d–4f, 5a and 5b). As with *esr1-1*, *esr1-2* plants exhibited increased basal expression of *LUCIFERASE* but not *GSTF8* (Fig 5c). To rule out the possibility that increased transgene activity in the *esr1* mutants contributes to *Fusarium* resistance, we inoculated SALK_095666 in the absence of the *GSTF8*:*LUC* transgene, and included control Col-0 plants. As with both *esr1-1* and *esr1-2* mutants, the SALK_09566 line also showed significantly increased resistance to *Fusarium* (Fig 5d and 5e). These results therefore confer a new role for At5g53060/ESR1, in mediating responses to biotic stress.

Of the other 50 constitutive *GSTF8*:*LUC* mutants isolated from our initial screen, we identified two further recessive alleles of *esr1* using the process of Next Generation Mapping (NGM) [37]. Using the NGM tool, SNPs were called against the TAIR10 genome reference and identified a region on chromosome 5 low in heterozygosity, incidentally mapping to the *ESR1/At5g53060* loci (S1 Fig). We found both mutants conferred increased resistance to *F*. *oxysporum* and through genetic complementation confirmed both mutants were indeed alleles of *esr1*, subsequently designating them as *esr1-3* and *esr1-4* (Fig 5f–5h). *esr1-3* confers a G2744A change at the second last exon/intron boundary (TGAGCAgtaagtt>TGAGCAataagtt), while *esr1-4* confers a G690A change at the last codon of the first exon causing a synonymous change (CAG>CAA, Q>Q) (Fig 5i). Processing of these mutated genes through the splice site prediction software NetGene2 (version 2.4; [55]) revealed that both mutants would encode miss-spliced transcripts with *esr1-3* losing the last donor splice site and *esr1-4* losing the first donor splice site.

### *esr1* mutants confer increased tolerance to heat stress

Three other mutant alleles of *At5g53060* have been independently identified through abiotic stress screens using salt, desiccation or cold-inducible promoters and confer increased or reduced tolerance to specific abiotic stresses. These are *Regulator of CBF gene expression 3* (*rcf3-1*, [26]), *Shiny1* (*shi1*, [27]), and *High Osmotic Stress Gene Expression 5* (*hos5-1*, [56]). To determine if *esr1-1* exhibited altered tolerance to abiotic stress we conducted heat tolerance assays described by [26] and found *esr1-1* also conferred increased tolerance to heat stress (S2 Fig). This was also confirmed for the insertional knockout mutant *esr1-2* (S2 Fig). To further characterise the role of At5g53060/ESR1 in temperature stress, we monitored *GSTF8*:*LUC* activity in wild-type and *esr1-1* seedlings over a 12 h time-course following heat stress. As with results following SA treatment (Fig 2), *GSTF8*:*LUC* activity increased more rapidly in *esr1-1* compared to wild-type seedlings (S2 Fig) suggesting ESR1 contributes to the repression of SA and heat induced stress responses.

### Whole transcriptome sequencing of *esr1-1* reveals altered expression of genes involved in biotic and abiotic stress responses

To uncover the possible direct or indirect targets of At5g53060/ESR1, we performed whole-transcriptome sequencing (RNA-seq) on three biological replicates of *esr1-1* and wild-type seedlings using the Illuminia HiSeq platform. We used un-treated seedlings as *GSTF8*:*LUC* is constitutively up-regulated in *esr1-1* under normal growing conditions (Fig 1a). Between 18.5 and 21.4 million paired-end reads (100 bp in length) per sample were mapped to the Arabidopsis TAIR10 exome reference sequence.

**Fig 6:**
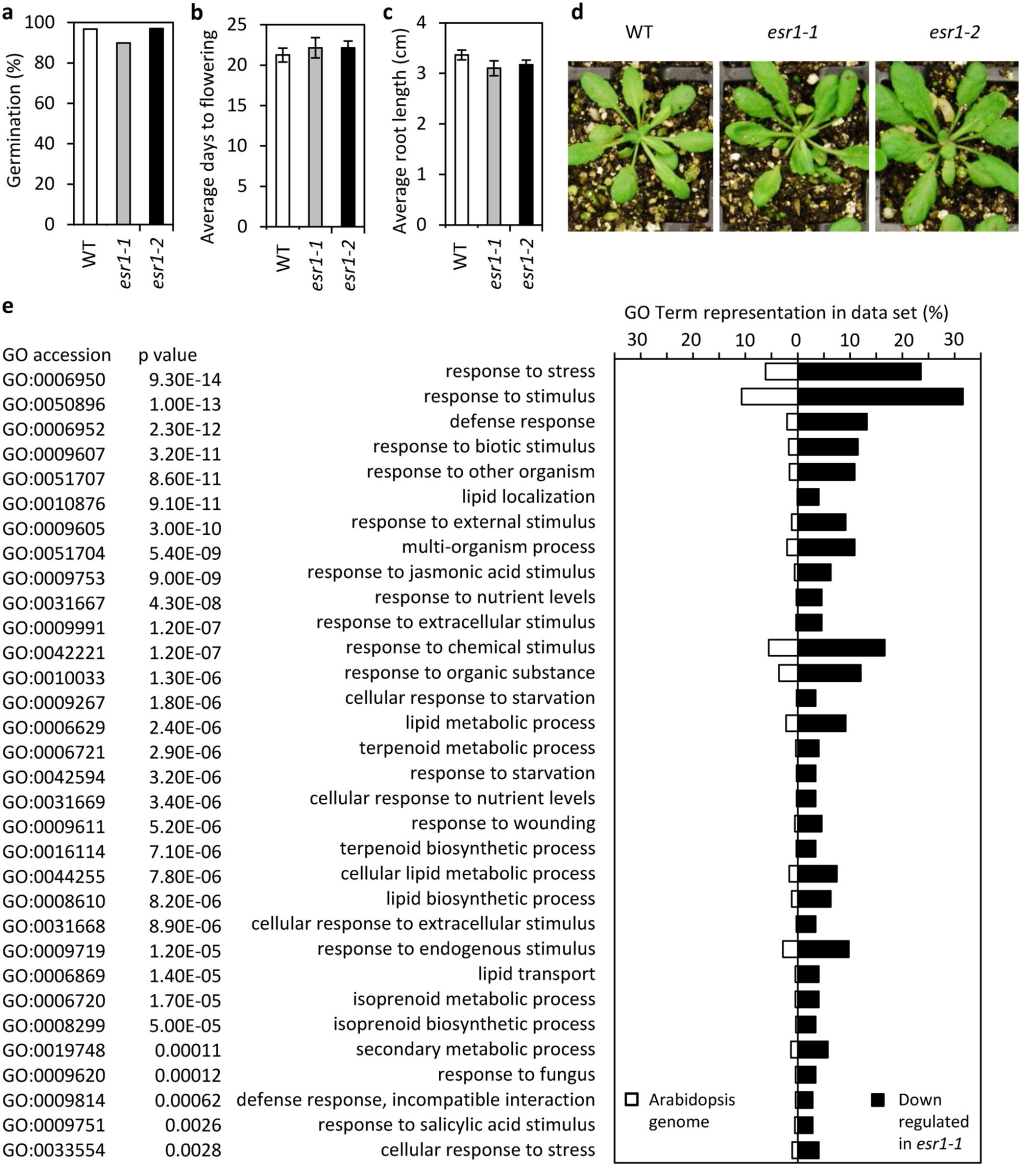
Significant enrichment of stress and defense associated biological process Gene Ontology (GO) terms in *esr1-1* down-regulated genes is not associated with developmental impairment. (a-d) Neither *esr1-1* nor *esr1-2* differ from wild-type in (a) germination, (b) flowering time, (c) root or (d) leaf development. (e) Genes significantly down-regulated ≥2-fold in *esr1-1* (compared to wild-type) were analyzed for enrichment of GO terms associated with biological processes. Shown are GO term representations in the *esr1-1* dataset compared to representation in the Arabidopsis genome. GO terms are ordered by p values adjusted by the False Discovery Rate.

Using the Cuffdiff tool within Cufflinks [49] we identified 1176 significantly differentially expressed genes between wild-type and *esr1-1* (Benjamini-Hochberg correction for multiple-testing based on a False Discovery Rate ≤0.05). Based on significant FPKMs (Fragments Per Kilobase of exon model per Million mapped fragments) fold changes, more transcripts were down-regulated in *esr1-1* (873) compared to up-regulated (303) (S2 and S3 Tables). To gain insight into the functions of these genes we performed Gene Ontology (GO) term enrichment analysis using agriGO v1.2 [57] with the default False Discovery Rate (p ≤0.05) determined p-value significance. Among the most significantly enriched biological processes from genes down-regulated in *esr1-1* were those involved in response to stress, biotic and abiotic stimuli, defense responses, wounding responses, and response to other organisms (bacteria and fungi) (S4 Table). Among the most significantly enriched biological processes from genes up-regulated in *esr1-1* were those involved in response to light, abiotic and hormone stimulus, cell death, signaling pathways and developmental processes (S5 Table). While *esr1-1* displays these significant changes in stress and defense responsive gene expression, interestingly neither *esr1-1* or *esr1-2* show obvious deleterious growth or developmental defects (Fig 6a–6d) often exhibited by Arabidopsis mutants with similar expression profiles [2, 58].

To consolidate a smaller list of differentially expressed genes for follow up qRT-PCR studies we sorted the gene lists for those with significantly altered expression greater than 2-fold from wild-type levels. This identified 48 genes up-regulated in *esr1-1* and 174 genes down-regulated (Tables 1 and 2). Although analysis of the 48 up-regulated genes did not reveal any specific GO term enrichment for biological processes, there was molecular function enrichment for nucleoside and nucleotide binding. The gene list included C-Terminal Domain Phosphatase-Like1 (CPL1) which can physically interact with At5g53060 [27, 56, 59] and was one of the most significantly up-regulated genes in *esr1-1*. Other genes with strong up-regulation were *At3g54160* an uncharacterised F-box/RNI-like protein, *SYNAPTOTAGMIN 2* (*SYTB*) a calcium-dependent lipid-binding protein involved in protein secretion, and *SUGAR TRANSPORTER 11* (*STP11*). Examination of these three genes and *CPL1* using qRT-PCR within a developmental time-course comparing wild-type versus *esr1-1* seedlings, including the 14-day old seedlings that were sampled for the RNAseq analysis, revealed their constitutive up-regulation at all time-points (S3 Fig).

**Table 1:** *esr1-1* ≥2-fold up-regulated genes.

AGI locus	symbol	fold change (*esr1-1*/WT)	FDR-adjusted p-value	AGI description
AT4G21670	CPL1	2.43	0	C-terminal domain phosphatase-like 1
AT1G17990		2.65	0	FMN-linked oxidoreductases superfamily protein
AT5G25280		**3.15**	0	serine-rich protein-related
AT5G25130	CYP71B12	2.16	3.6E-15	cytochrome P450, family 71, subfamily B, polypeptide 12
AT2G01860	EMB975	2.48	3.3E-11	Tetratricopeptide repeat (TPR)-like superfamily protein, EMBRYO DEFECTIVE 975
AT1G03990		**4.15**	1.9E-09	Long-chain fatty alcohol dehydrogenase family protein
AT5G66520		2.39	1.2E-08	Tetratricopeptide repeat (TPR)-like superfamily protein
AT1G19340		2.75	2.1E-06	Methyltransferase MT-A70 family protein
AT3G54160		**14.45**	2.4E-05	RNI-like superfamily protein
AT5G44120	CRA1	2.07	8.3E-05	RmlC-like cupins superfamily protein, CRUCIFERINA
AT2G36790	UGT73C6	2.07	1.2E-04	UDP-glucosyl transferase 73C6
AT1G61400		2.78	1.5E-04	S-locus lectin protein kinase family protein
AT5G11470		2.35	2.5E-04	bromo-adjacent homology (BAH) domain-containing protein
AT2G42730		2.34	2.5E-04	F-box family protein
AT5G44980		**3.55**	3.2E-04	F-box/RNI-like/FBD-like domains-containing protein
AT1G50790		2.55	3.8E-04	Plant mobile domain protein family
AT1G61500		2.09	7.6E-04	S-locus lectin protein kinase family protein
AT1G77960		**3.26**	1.3E-03	Unknown
AT2G30505		2.91	1.3E-03	Late embryogenesis abundant (LEA) hydroxyproline-rich glycoprotein family
AT3G57580		2.11	2.0E-03	F-box and associated interaction domains-containing protein
AT3G53680		2.02	2.9E-03	Acyl-CoA N-acyltransferase with RING/FYVE/PHD-type zinc finger domain
AT1G52990		**7.45**	2.9E-03	thioredoxin family protein
AT1G75110	RRA2	2.11	4.2E-03	Nucleotide-diphospho-sugar transferase family protein, REDUCED RESIDUAL ARABINOSE 2
AT3G26550		2.08	5.4E-03	Cysteine/Histidine-rich C1 domain family protein
AT4G03440		2.03	5.4E-03	Ankyrin repeat family protein
AT4G28520	CRU3	2.01	5.6E-03	cruciferin 3
AT3G44713		3.25	5.7E-03	Unknown
AT1G13609		2.16	6.3E-03	Defensin-like (DEFL) family protein
AT2G07732		2.05	6.5E-03	Ribulose bisphosphate carboxylase large chain, catalytic domain
AT1G12700		2.07	7.0E-03	ATP binding;nucleic acid binding;helicases
AT3G21370	BGLU19	**3.89**	7.6E-03	beta glucosidase 19
AT5G37750		**3.70**	8.8E-03	Chaperone DnaJ-domain superfamily protein
AT1G51520		2.06	9.4E-03	RNA-binding (RRM/RBD/RNP motifs) family protein
AT5G37400		2.15	9.4E-03	Family of unknown function (DUF577)
AT3G18970	MEF20	2.18	1.0E-02	mitochondrial editing factor 20
AT1G20080	SYTB	**7.60**	1.1E-02	Calcium-dependent lipid-binding (CaLB domain) family protein
AT5G23270	STP11	**6.74**	1.2E-02	sugar transporter 11
AT2G19910		2.15	1.3E-02	RNA-dependent RNA polymerase family protein
AT3G47090		2.09	1.5E-02	Leucine-rich repeat protein kinase family protein
AT5G38040		2.15	1.8E-02	UDP-Glycosyltransferase superfamily protein
AT5G23600		2.21	2.0E-02	RNA 2'-phosphotransferase, Tpt1 / KptA family
AT3G46370		2.13	2.3E-02	Leucine-rich repeat protein kinase family protein
AT4G16940		2.12	2.6E-02	Disease resistance protein (TIR-NBS-LRR class) family
AT5G43840	HSFA6A	2.74	3.2E-02	heat shock transcription factor A6A
AT5G66960		2.02	3.5E-02	Prolyl oligopeptidase family protein
AT4G38010		2.59	4.0E-02	Pentatricopeptide repeat (PPR-like) superfamily protein
AT1G58320		2.14	4.0E-02	PLAC8 family protein
AT4G10600		2.66	4.1E-02	RING/FYVE/PHD zinc finger superfamily protein

Fold change based on FPKMs from 3 biological replicates. Significance based on Benjamini-Hochberg correction for multiple-testing, P≤0.05 adjusted by the False Discovery Rate. Values in bold are ≥3-fold changes.

**Table 2:** *esr1-1* ≥2-fold down-regulated genes.

AGI locus	symbol	fold change (*esr1-1*/WT)	FDR-adjusted p-value	AGI description
AT3G49620	DIN11	**15.9**	0	2-oxoglutarate (2OG) and Fe(II)-dependent oxygenase protein, DARK INDUCBILE 11
AT2G39030	NATA1	**8.6**	0	Acyl-CoA N-acyltransferases (NAT) superfamily protein
AT5G42600	MRN1	**6.1**	0	marneral synthase
AT3G12740	ALIS1	**4.8**	0	ALA-interacting subunit 1
AT4G15210	BAM5	**4.5**	0	beta-amylase 5
AT2G39330	JAL23	**3.8**	0	jacalin-related lectin 23
AT3G57260	BGL2	**3.4**	0	beta-1,3-glucanase 2
AT3G25830	TPS-CIN	**3.3**	0	terpene synthase-like sequence-1,8-cineole
AT1G33960	AIG1	**3.3**	0	P-loop containing nucleoside triphosphate hydrolases superfamily protein, AVRRPT2-INDUCED GENE 1
AT2G24850	TAT3	**3.1**	0	tyrosine aminotransferase 3
AT1G45201	TLL1	2.9	0	triacylglycerol lipase-like 1
AT5G20150	SPX1	2.9	0	SPX domain gene 1
AT2G43510	TI1	2.6	0	trypsin inhibitor protein 1
AT2G39310	JAL22	2.5	0	jacalin-related lectin 22
AT3G04720	PR4	2.4	0	pathogenesis-related 4
AT1G19670	CLH1	2.3	0	chlorophyllase 1
AT5G24770	VSP2	2.3	0	vegetative storage protein 2
AT5G48850	ATSDI1	2.1	0	Tetratricopeptide repeat (TPR)-like superfamily protein, SULPHUR DEFICIENCY-INDUCED 1
AT3G45140	LOX2	2.1	0	lipoxygenase 2
AT1G73260	KTI1	2.0	0	kunitz trypsin inhibitor 1
AT4G22517		**3.5**	3.6E-15	Bifunctional inhibitor/lipid-transfer protein/seed storage 2S albumin superfamily protein
AT2G43530		2.8	3.6E-15	Scorpion toxin-like knottin superfamily protein
AT5G24780	VSP1	2.5	7.2E-15	vegetative storage protein 1
AT3G21500	DXPS1	**6.7**	1.8E-14	1-deoxy-D-xylulose 5-phosphate synthase 1
AT3G49580	LSU1	2.5	3.4E-14	response to low sulfur 1
AT5G42590	CYP71A16	2.4	5.7E-14	cytochrome P450, family 71, subfamily A, polypeptide 16
AT4G12470	AZI1	2.2	1.8E-13	azelaic acid induced 1
AT1G27020		2.4	1.9E-13	Unknown
AT2G14610	PR1	2.3	2.2E-13	pathogenesis-related gene 1
AT5G20790		2.8	1.2E-12	Unknown
AT3G02040	SRG3	2.1	1.2E-12	senescence-related gene 3
AT4G22470		**3.7**	1.7E-12	protease inhibitor/seed storage/lipid transfer protein (LTP) family protein
AT2G29900	PS2	2.4	9.0E-12	Presenilin-2
AT1G61120	TPS04	**7.2**	1.8E-11	terpene synthase 04
AT4G25000	AMY1	**4.7**	3.3E-11	alpha-amylase-like
AT3G44860	FAMT	**3.8**	5.6E-11	farnesoic acid carboxyl-O-methyltransferase
AT5G10380	RING1	2.7	6.8E-11	RING/U-box superfamily protein
AT1G17710		2.6	9.0E-11	Pyridoxal phosphate phosphatase-related protein
AT2G29350	SAG13	2.4	1.4E-10	senescence-associated gene 13
AT1G15520	PDR12	2.6	1.9E-10	pleiotropic drug resistance 12
AT5G10760		3.1	3.7E-10	Eukaryotic aspartyl protease family protein
AT3G26830	PAD3	2.2	1.5E-09	Cytochrome P450 superfamily protein, PHYTOALEXIN DEFICIENT 3
AT1G69880	TH8	**3.1**	2.5E-09	thioredoxin H-type 8
AT3G25760	AOC1	2.1	2.9E-09	allene oxide cyclase 1
AT1G14250		2.5	6.0E-09	GDA1/CD39 nucleoside phosphatase family protein
AT4G37990	ELI3-2	**6.3**	7.5E-09	elicitor-activated gene 3–2
AT3G55970	JRG21	**4.1**	1.5E-08	jasmonate-regulated gene 21
AT2G18660	PNP-A	**3.8**	4.2E-08	plant natriuretic peptide A
AT3G17790	PAP17	2.3	4.4E-08	purple acid phosphatase 17
AT5G23980	FRO4	2.6	5.0E-08	ferric reduction oxidase 4
AT2G16005		2.2	8.2E-08	MD-2-related lipid recognition domain-containing protein
AT3G26840		2.3	8.6E-08	Esterase/lipase/thioesterase family protein
AT3G28540		2.2	1.3E-07	P-loop containing nucleoside triphosphate hydrolases superfamily protein
AT4G21830	MSRB7	2.6	2.5E-07	methionine sulfoxide reductase B7
AT2G14560	LURP1	2.9	2.5E-07	LATE UPREGULATED IN RESPONSE TO HYALOPERONOSPORA PARASITICA
AT1G10585		**3.8**	2.8E-07	basic helix-loop-helix (bHLH) DNA-binding superfamily protein
AT2G39510		2.0	3.0E-07	nodulin MtN21 /EamA-like transporter family protein
AT3G05630	PLDP2	2.3	3.0E-07	phospholipase D P2
AT3G46900	COPT2	2.6	3.9E-07	copper transporter 2
AT4G21326	SBT3.12	**3.1**	4.6E-07	subtilase 32
AT2G44460	BGLU28	2.4	6.7E-07	beta glucosidase 28
AT5G42580	CYP705A12	2.1	7.2E-07	cytochrome P450, family 705, subfamily A, polypeptide 12
AT5G23990	FRO5	**5.6**	8.1E-07	ferric reduction oxidase 5
AT4G32810	CCD8	2.9	1.4E-06	carotenoid cleavage dioxygenase 8
AT5G20710	BGAL7	2.0	1.4E-06	beta-galactosidase 7
AT4G17470		**3.6**	2.0E-06	alpha/beta-Hydrolases superfamily protein
AT3G05400		2.0	2.2E-06	Major facilitator superfamily protein
AT1G19380		2.8	2.3E-06	Protein of unknown function (DUF1195)
AT1G32350	AOX1D	**3.1**	2.9E-06	alternative oxidase 1D
AT5G04120		2.2	5.8E-06	Phosphoglycerate mutase family protein
AT2G29470	GSTU3	**4.5**	7.4E-06	glutathione S-transferase tau 3
AT3G22910		2.5	8.6E-06	ATPase E1–E2 type family protein / haloacid dehalogenase-like hydrolase family protein
AT1G76960		2.1	1.2E-05	Unknown
AT4G21680	NRT1.8	**3.5**	1.3E-05	NITRATE TRANSPORTER 1.8
AT5G45430		2.1	1.5E-05	Protein kinase superfamily protein
AT3G45130	LAS1	**3.0**	2.6E-05	lanosterol synthase 1
AT1G17420	LOX3	2.3	2.8E-05	lipoxygenase 3
AT2G43020	PAO2	**13.7**	3.4E-05	polyamine oxidase 2
AT1G61800	GPT2	2.0	4.8E-05	glucose-6-phosphate/phosphate translocator 2
AT1G12030		**3.3**	5.6E-05	Protein of unknown function (DUF506)
AT1G02470		**3.7**	5.6E-05	Polyketide cyclase/dehydrase and lipid transport superfamily protein
AT1G07620	ATOBGM	2.3	5.7E-05	GTP-binding protein Obg/CgtA
AT2G11810	MGDC	2.3	6.3E-05	monogalactosyldiacylglycerol synthase type C
AT5G08760		2.1	6.9E-05	Unknown
AT1G07400		2.1	7.4E-05	HSP20-like chaperones superfamily protein
AT1G60110		**3.1**	1.1E-04	Mannose-binding lectin superfamily protein
AT3G45860	CRK4	2.1	1.1E-04	cysteine-rich RLK (RECEPTOR-like protein kinase) 4
AT4G12490		2.2	1.3E-04	Bifunctional inhibitor/lipid-transfer protein/seed storage 2S albumin superfamily protein
AT4G11890		2.6	1.8E-04	Protein kinase superfamily protein
AT4G04490	CRK36	2.5	2.3E-04	cysteine-rich RLK (RECEPTOR-like protein kinase) 36
AT5G24200		2.8	2.6E-04	alpha/beta-Hydrolases superfamily protein
AT5G44420	PDF1.2	**3.6**	2.6E-04	plant defensin 1.2
AT2G45130	SPX3	2.8	2.8E-04	SPX domain gene 3
AT2G04450	NUDT6	2.2	2.9E-04	nudix hydrolase homolog 6
AT4G24000	CSLG2	2.8	3.0E-04	cellulose synthase like G2
AT2G36970		2.1	4.0E-04	UDP-Glycosyltransferase superfamily protein
AT2G26400	ARD3	**3.0**	5.0E-04	acireductone dioxygenase 3
AT4G24340		2.5	5.1E-04	Phosphorylase superfamily protein
AT5G39520		**3.2**	8.4E-04	Protein of unknown function (DUF1997)
AT1G05660		2.7	8.6E-04	Pectin lyase-like superfamily protein
AT1G52100		2.5	9.6E-04	Mannose-binding lectin superfamily protein
AT2G39040		2.5	1.1E-03	Peroxidase superfamily protein
AT1G73805		2.1	1.2E-03	Calmodulin binding protein-like
AT3G52970	CYP76G1	2.3	1.3E-03	cytochrome P450, family 76, subfamily G, polypeptide 1
AT4G36700		2.8	1.4E-03	RmlC-like cupins superfamily protein
AT3G51450		2.1	1.7E-03	Calcium-dependent phosphotriesterase superfamily protein
AT3G05650	RLP32	2.0	1.7E-03	receptor like protein 32
AT2G45570	CYP76C2	2.2	1.8E-03	cytochrome P450, family 76, subfamily C, polypeptide 2
AT1G71400	RLP12	2.2	1.9E-03	receptor like protein 12
AT2G22860	PSK2	**3.1**	3.2E-03	phytosulfokine 2 precursor
AT4G21840	MSRB8	2.3	3.2E-03	methionine sulfoxide reductase B8
AT1G23730	BCA3	2.6	3.4E-03	beta carbonic anhydrase 3
AT5G46050	PTR3	2.4	3.5E-03	peptide transporter 3
AT3G43110		2.3	3.6E-03	Unknown
AT2G40330	PYL6	2.4	4.3E-03	PYR1-like 6
AT5G52760		2.4	4.8E-03	Copper transport protein family
AT1G15540		2.6	4.9E-03	2-oxoglutarate (2OG) and Fe(II)-dependent oxygenase superfamily protein
AT1G80160		2.1	5.6E-03	Lactoylglutathione lyase / glyoxalase I family protein
AT1G51820		2.2	5.9E-03	Leucine-rich repeat protein kinase family protein
AT3G59370		2.0	6.0E-03	Vacuolar calcium-binding protein-related
AT3G09405		2.9	6.2E-03	Pectinacetylesterase family protein
AT3G46660	UGT76E12	2.1	6.2E-03	UDP-glucosyl transferase 76E12
AT3G04530	PPCK2	2.0	6.5E-03	phosphoenolpyruvate carboxylase kinase 2
AT1G08310		2.1	7.3E-03	alpha/beta-Hydrolases superfamily protein
AT2G38240		2.9	7.5E-03	2-oxoglutarate (2OG) and Fe(II)-dependent oxygenase superfamily protein
AT1G35230	AGP5	2.3	7.7E-03	arabinogalactan protein 5
AT2G34655		2.3	8.8E-03	Unknown
AT3G13840		2.3	9.0E-03	GRAS family transcription factor
AT2G26010	PDF1.3	2.2	9.1E-03	plant defensin 1.3
AT5G43690		2.9	9.6E-03	P-loop containing nucleoside triphosphate hydrolases superfamily protein
AT4G37700		2.3	9.7E-03	Unknown
AT2G14210	AGL44	2.9	1.0E-02	AGAMOUS-like 44
AT3G06435		2.1	1.0E-02	Expressed protein
AT5G14180	MPL1	2.6	1.1E-02	Myzus persicae-induced lipase 1
AT1G52890	NAC019	2.7	1.1E-02	NAC domain containing protein 19
AT1G19200		2.0	1.2E-02	Protein of unknown function (DUF581)
AT3G57460		6.7	1.2E-02	catalytics;metal ion binding
AT1G01680	PUB54	2.1	1.2E-02	plant U-box 54
AT1G33950		6.6	1.3E-02	Avirulence induced gene (AIG1) family protein
AT3G24310	MYB305	2.5	1.3E-02	myb domain protein 305
AT4G24110		2.1	1.6E-02	Unknown
AT4G33560		2.0	1.6E-02	Wound-responsive family protein
AT3G12070	RGTB2	2.4	1.8E-02	RAB geranylgeranyl transferase beta subunit 2
AT1G73325		6.3	1.8E-02	Kunitz family trypsin and protease inhibitor protein
AT1G54020		5.2	1.9E-02	GDSL-like Lipase/Acylhydrolase superfamily protein
AT2G34350		2.7	2.0E-02	Nodulin-like / Major Facilitator Superfamily protein
AT3G46090	ZAT7	3.1	2.0E-02	C2H2 and C2HC zinc fingers superfamily protein
AT2G32660	RLP22	2.1	2.1E-02	receptor like protein 22
AT5G27060	RLP53	2.4	2.2E-02	receptor like protein 53
AT4G27160	SESA3	28.7	2.3E-02	seed storage albumin 3
AT1G17380	JAZ5	2.2	2.4E-02	jasmonate-zim-domain protein 5
AT1G72260	THI2.1	5.4	2.4E-02	thionin 2
AT2G37740	ZFP10	2.7	2.4E-02	zinc-finger protein 10
AT1G32960	SBT3.3	2.8	2.5E-02	Subtilase family protein
AT1G71200		2.8	2.7E-02	basic helix-loop-helix (bHLH) DNA-binding superfamily protein
AT1G65570		2.9	2.8E-02	Pectin lyase-like superfamily protein
AT2G14620	XTH10	2.1	3.1E-02	xyloglucan endotransglucosylase/hydrolase 10
AT1G36622		2.0	3.1E-02	Unknown
AT1G19610	PDF1.4	3.4	3.2E-02	Arabidopsis defensin-like protein
AT1G28370	ERF11	2.6	3.2E-02	ERF domain protein 11
AT1G63055		7.9	3.3E-02	Unknown
AT3G04510	LSH2	2.9	3.4E-02	Protein of unknown function (DUF640)
AT4G13410	ATCSLA15	2.3	3.6E-02	Nucleotide-diphospho-sugar transferases superfamily protein
AT5G44430	PDF1.2c	12.8	3.6E-02	plant defensin 1.2C
AT5G43290	WRKY49	3.5	3.9E-02	WRKY DNA-binding protein 49
AT5G55410		3.7	4.0E-02	Bifunctional inhibitor/lipid-transfer protein/seed storage 2S albumin superfamily protein
AT4G01630	EXPA17	2.1	4.1E-02	expansin A17
AT2G45760	BAP2	2.7	4.1E-02	BON association protein 2
AT4G04500	CRK37	2.0	4.4E-02	cysteine-rich RLK (RECEPTOR-like protein kinase) 37
AT3G03480	CHAT	3.5	4.5E-02	acetyl CoA:(Z)-3-hexen-1-ol acetyltransferase
AT4G28085		2.7	4.7E-02	Unknown
AT1G69720	HO3	3.4	4.7E-02	heme oxygenase 3
AT5G46350	WRKY8	2.1	4.8E-02	WRKY DNA-binding protein 8
AT5G13220	JAZ10	2.9	4.9E-02	jasmonate-zim-domain protein 10

Fold change based on FPKMs from 3 biological replicates. Significance based on Benjamini-Hochberg correction for multiple-testing, P≤0.05 adjusted by the False Discovery Rate. Values in bold are ≥3-fold changes.

**Fig 7:**
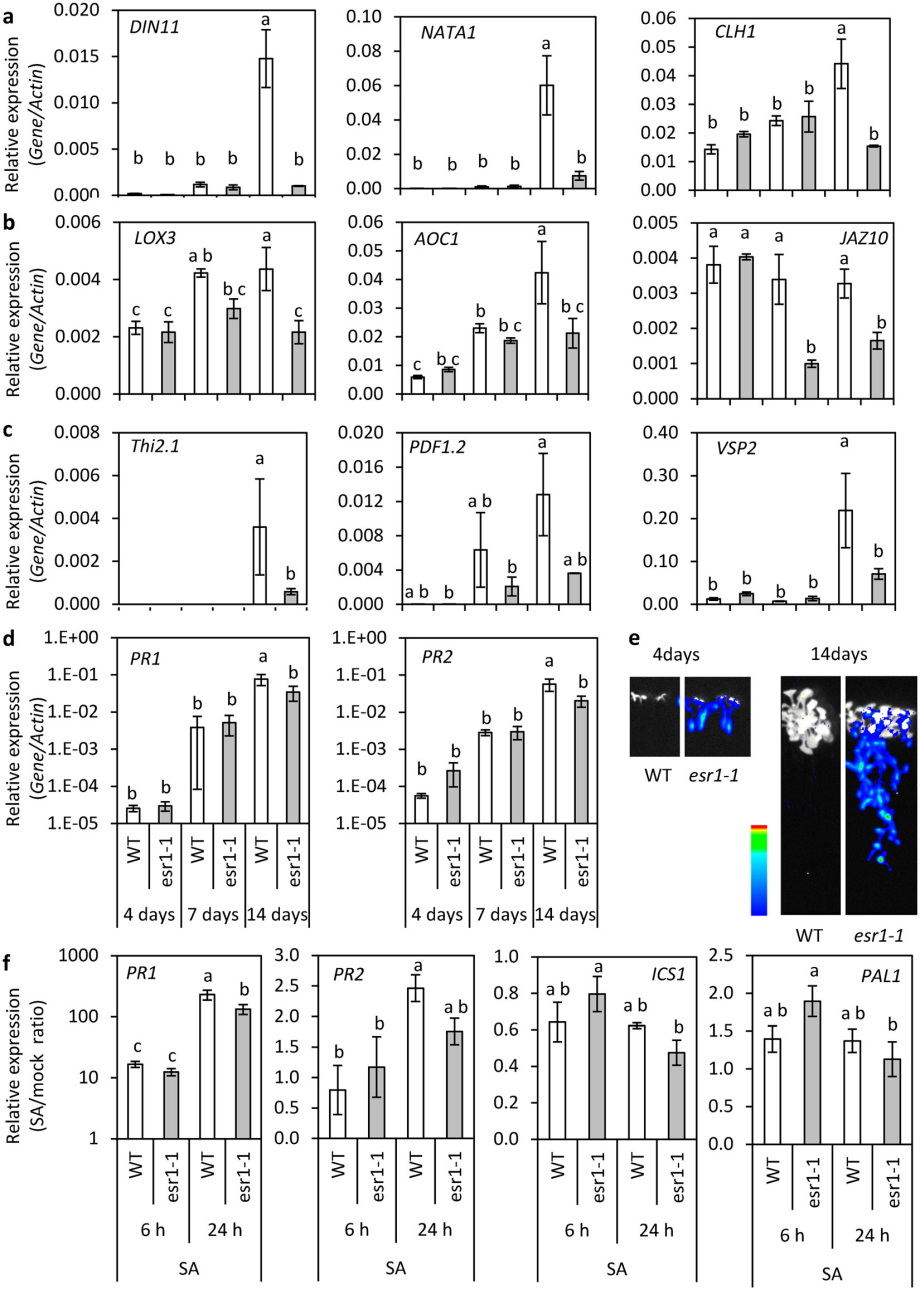
Repression of JA-mediated gene expression in *esr1-1* increases with age. (a-c) Expression of significantly up-regulated (a) novel RNA-seq identified, (b) JA-biosynthesis and signalling, (c) JA-regulated defense and wound-responsive genes, and (d) SA-regulated defense genes in *esr1-1* compared to wild-type (WT) seedlings as determined by qRT-PCR. Shown are values from 4, 7 and 14 day old seedlings (values are averages ± SE of 3 biological replicates consisting of pools of 20 seedlings, *P*<0.05, all pairs Student’s t-test). Gene expression levels are relative to the internal control *β-actin* genes. (e) Increasing *GSTF8*:*LUC* activity in *esr1-1* seedlings during early development. (f) Fold changes in SA-marker genes in WT and *esr1-1* seedlings 6 and 24 hours post SA treatment. Shown are values from 12 day old seedlings (values are averages ± SE of 3 biological replicates consisting of pools of 20–30 seedlings, *P*<0.05, all pairs Student’s t-test). Transcript levels of each gene of interest following SA treatment were normalised against the internal control *β-actin* genes and expressed relative to the normalised levels in mock-treated WT or *esr1-1* seedlings.

We next examined the list of 174 genes that were significantly down-regulated in *esr1-1* >2-fold over wild-type (Table 2) for GO term enrichment. Amongst the most significantly enriched biological process GO terms were those involved in responses to stress, defense, biotic stimulus, other organisms, jasmonic acid (JA) including JA-biosynthesis and-signalling, fungus, wounding, SA, chemical stimulus and responses to starvation and nutrient levels (Fig 6e). As with the up-regulated *esr1-1* dataset, to confirm our RNAseq data we examined the expression of several down-regulated genes over a developmental time-course. This included the highly down-regulated genes *DARK INDUCIBLE 11* (*DIN11*) encoding a 2-oxoglutarate (2OG) and Fe(II)-dependent oxygenase, *At2g39030/NATA1* encoding an Acyl-CoA N-acyltransferase (NAT) superfamily protein with roles in pathogen resistance [60], as well as *CHLOROPHYLLASE 1*/*CORONATINE-INDUCED PROTEIN 1* (*CLH1*/*CORI1*) which has roles in several of the enriched biological process GO categories including defense responses, response to fungus and JA-signalling. No significant difference in *DIN11*, *NATA1* or *CLH1* expression was observed in 4- or 7-day old seedlings however, as in the RNAseq dataset they were highly down-regulated in 14-day old seedlings (Fig 7a).

### At5g53060/ESR1 affects basal JA-mediated responses involved in defense but not growth and development

A role for At5g53060 in JA-responses to our knowledge has not been described before, and as the down-regulated *esr1-1* gene list was enriched for genes with roles in these processes including defense and biotic stimulus (response to fungus and wounding), we were interested to dissect this further. We first examined the expression of representative JA-biosynthesis, signalling, and JA-regulated defense genes. Using qRT-PCR, the *LIPOXYGENASE 3* (*LOX3*) and *ALLENE OXIDE CYCLASE 1* (*AOC1*) genes involved in JA-biosynthesis, and *JASMONATE-ZIM-DOMAIN PROTEIN 10* (*JAZ10*) involved in repression of JA-responses were down-regulated in *esr1-1* in both 7-and 14-day old seedlings (Fig 7b) and were identified in the RNAseq dataset as down-regulated genes (Table 2). The down-regulation of these genes suggests an overall down-regulation of JA-signalling processes in *esr1-1* as their expression is in part regulated through JA-feedback loops [61–63]. In 14-day old seedlings the JA-regulated defense and wound marker genes analysed were all down-regulated in *esr1-1* compared to wild-type seedlings (Fig 7c). The expression of these marker genes in 4- or 7-day old seedlings was either lowly expressed or not detectable by qRT-PCR. Overall expression patterns in wild-type seedlings highlighted a trend in increasing expression from 4- to 14-days. Examination of these genes in publically available, developmental series transcriptome datasets (Genevestigator; [64]) also revealed similar gene expression profiles in wild-type plants (data not shown). *GSTF8*:*LUC* activity also increases in *esr1-1* seedlings over this timeframe (Fig 7e). Together, these results suggest At5g53060/ESR1 has a negative effect on *GSTF8*:*LUC* activity and a positive effect on the regulation of JA-mediated genes during early development.

In addition to roles in defense, JA also affects fertility, root growth and development [65–69]. However, neither *esr1-1* nor *esr1-2* are impaired in these processes (Fig 6a–6d). We also found the *esr1* mutants were not affected in JA-sensitivity as determined by methyl jasmonate (MeJA) root inhibition assays (S4 Fig). This suggests At5g53060/ESR1 functions in activation of a subset of JA-mediated responses.

It is well known that antagonistic interactions occur between some aspects of JA and SA signalling (reviewed in [6, 8, 29, 70]. We therefore analysed expression of the SA-marker genes *PR1* and *PR2* in wild-type and *esr1-1* seedlings to determine if repression of JA-regulated genes in *esr1-1* was due to up-regulated SA-mediated signalling as is suggested by increased *GSTF8*:*LUC* activity and *GSTF8* expression in *esr1-1* following SA treatment (Fig 2). There was no significant difference in *PR1* or *PR2* expression between wild-type and *esr1-1* at 4 and 7 days of age, but their expression was significantly reduced in *esr1-1* at 14 days as was also detected by RNAseq (Fig 7d, Table 2). *PR1*, but not *PR2* expression, was also down-regulated in *esr1-1* following SA treatment (Fig 7f). To determine if other aspects of SA-signalling where altered in *esr1-1*, we determined expression of the *ISOCHORISMATE SYNTHASE1* (*ICS1*) and *PHENYLALANINE AMMONIA LYASE* (*PAL1*) genes involved in SA-biosynthesis (reviewed by [71]). Neither of these genes were significantly altered in expression suggesting ESR1 functions specifically in JA-signalling and down-regulation of *PR1* expression is due to non-SA-mediated processes.

### At5g53060/ESR1 is required for full activation of a subset of JA-regulated genes

**Fig 8:**
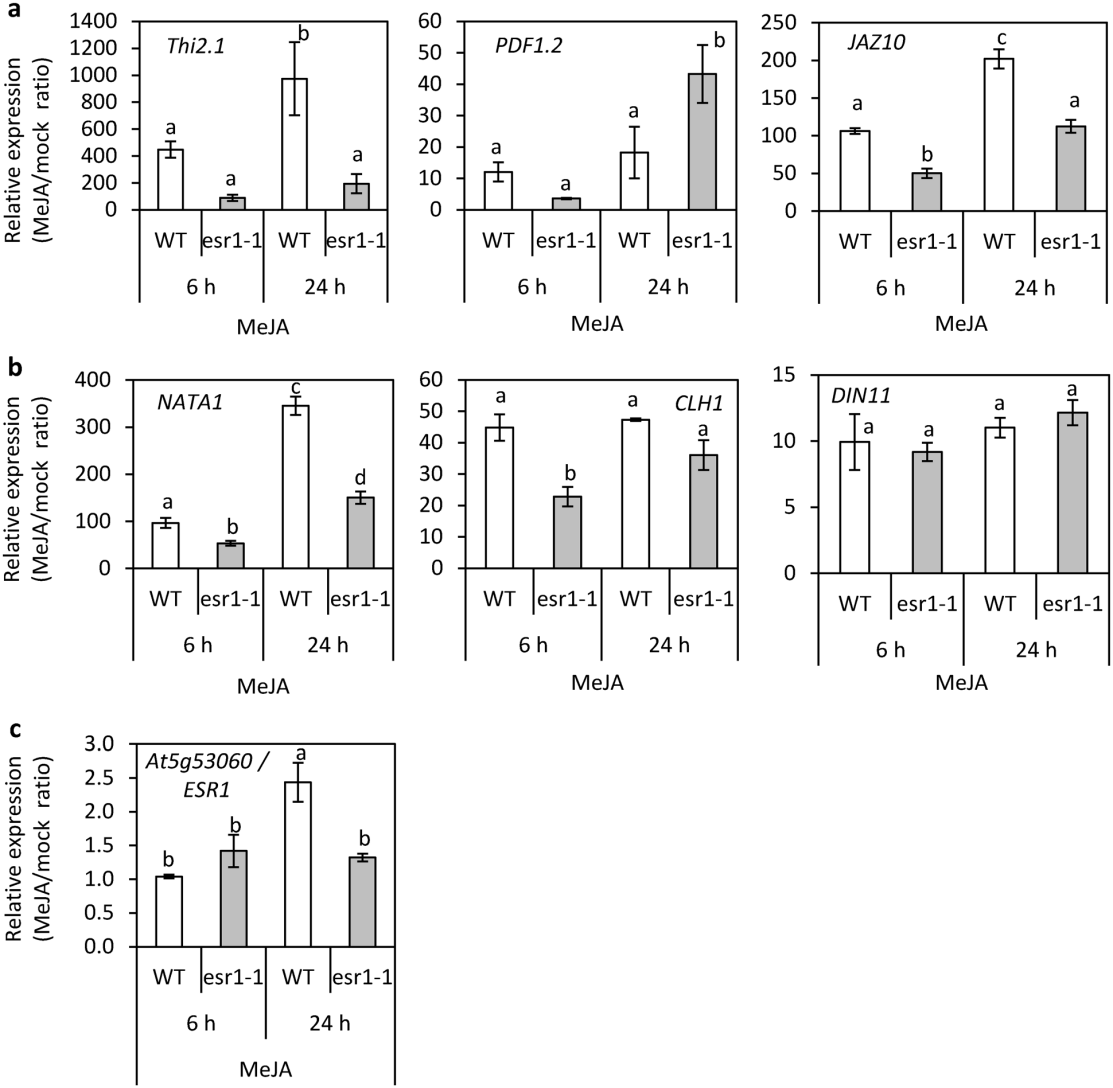
*esr1-1* represses a subset of JA-induced gene expression. (a-c) Fold changes in relative transcript abundance of (a) JA-regulated defense and signalling marker genes, (b) RNA-seq identified genes, and (c) *ESR1* in wild-type (WT) and *esr1-1* seedlings 6 and 24 hours post MeJA treatment. Shown are values from 12 day old seedlings (values are averages ± SE of 3 biological replicates consisting of pools of 20–30 seedlings, *P*<0.05, all pairs Student’s t-test). Transcript levels of each gene of interest following MeJA treatment were normalised against the internal control *β-actin* genes and expressed relative to the normalised levels in mock-treated WT or *esr1-1* seedlings.

Other mutants with reduced basal JA-biosynthesis or JA-regulated defense gene expression and exhibiting increased resistance to *Fusarium oxysporum* include *coi1* (*coronatine insenstive1*) and *pft1/med25* (*phytochrome and flowering time1*) [41, 45]. Expression of JA-regulated genes in these two mutants are also reduced following MeJA treatment. To determine if At5g53060/ESR1 affected the JA-inducibility of JA-regulated genes and other genes down-regulated in *esr1-1*, we examined the expression of *Thi2*.*1*, *PDF1*.*2*, *JAZ10*, *NATA1*, *CLH1* and *DIN11* in *esr1-1* and wild-type plants following MeJA or a mock treatment. As expected, MeJA treatment strongly induced *Thi2*.*1*, *PDF1*.*2* and *JAZ10* expression in wild-type plants relative to the mock-treated wild-type plants (Fig 8a). Expression of these genes was also induced by MeJA in *esr1-1* however, *Thi2*.*1* and *JAZ10* expression was 5-fold and 2-fold less respectively in *esr1-1* compared to wild-type levels at 6 and 12 hours post treatment. *PDF1*.*2* expression was also reduced in *esr1-1* at 6 hours but increased above wild-type levels at 24 hours. We next examined *NATA1*, *CLH1* and *DIN11* expression and found *esr1-1* had reduced induction of *NATA1* and *CLH1*, but did not affect the MeJA-induced expression of *DIN11* (Fig 8b). We also found *ESR1* expression is MeJA-inducible (Fig 8c). Combined, these results suggest ESR1 affects components of JA-signalling.

## Discussion

In a forward genetic screen using the defense and stress responsive *GSTF8* promoter, we isolated several alleles of the constitutive *GSTF8*:*LUC* expression mutant *esr1* encoding the KH-domain containing RNA-binding protein At5g53060. We identify At5g53060 as a susceptibility gene for *F*. *oxysporum* disease symptom development and a requirement for full-activation of components of JA-mediated gene expression. Four independent mutants of *At5g53060* termed *esr1-1*, *esr1-2*, *esr1-3* and *esr1-4* displayed increased resistance to *F*. *oxysporum* and define new roles for plant KH-domain containing proteins, linking At5g53060 to biotic stress and JA-mediated defense responses.

In plants the most widely spread RNA-binding domains are the RNA Recognition Motif, the heterogeneous nuclear ribonucleoprotein K (hnRNP K) homology (KH), and Pentatricopeptide Repeat (PPR) [10, 72]. Most plant RNA-binding proteins contain one or more of these domains, often combined with multiple auxiliary domains involved in protein-protein interactions or protein targeting, or other RNA-binding domains. An InterPro Scan of At5g53060 for known protein signatures only identified its five KH-domains (data not shown). KH-domain proteins typically contain more than one KH-domain where they can function independently or co-operatively to bind RNA or ssDNA [54]. The KH-domain was first identified in the human hnRNP K protein and is characterised by a conserved VIGXXGXXI sequence in the middle of the ~60 amino acid domain [11, 54]. In addition to At5g53060, only three other of the 26 predicted Arabidopsis KH proteins have been functionally characterised and these have roles in flowering, floral morphogenesis, and vegetative and reproductive development [73–75]. Unlike mutants of these *KH* genes, we found neither *esr1-1* nor *esr1-2* exhibited observable differences in flowering, growth or development (Fig 6a–6d).

In plants, RNA-binding proteins have been identified as regulators of floral transition, floral patterning, circadian rhythm, chromatin modification, ABA signalling and mediators of abiotic stress responses such as to dehydration, drought, flooding, salinity, cold and heat ([9, 10] and references within). However, few RNA-binding proteins have been characterized for roles in plant immunity [76, 77]. Examples include the RNA Recognition Motif containing proteins ATBRN1/ATRBP-DR1 and GLYCINE RICH PROTEIN 7 regulators of resistance against *Pseudomonas syringae* pv. *tomato* DC3000 [78, 79], and the double stranded RNA-binding domain proteins DICER LIKE2 and DICER LIKE4 involved in viral defense ([76, 80] and references within). Some RNA-binding proteins directly target pathogen RNA to control infection. For example PATHOGENESIS RELATED PROTEIN 10 members such as the cotton PR10 have ribonuclease activity [1, 81]. In addition to our findings on At5g53060, the only other plant KH-domain containing protein characterised for a role in plant immunity is BINDING TO TOMV RNA 1 (BTR1, At5g04430) [82] which functions by directly binding to Tomato Mosaic Virus (TOMV) RNA and preventing viral multiplication.

Through non-biased whole transcriptome sequencing we found *esr1-1* plants exhibited significant down-regulation of genes involved in responses to defense, biotic stimulus, fungus, wounding, and JA including JA-biosynthesis and-signalling (Fig 6). JA has roles in defense against specific microbial pathogens and insect pests, wounding responses, and roles in developmental processes such as fertility and root growth [65, 67–69]. The *esr1* mutants were not affected in latter responses but were affected in pathogen defense. Further, in addition to repression of basal JA-mediated gene expression, the MeJA inducibility of most JA-regulated genes tested were also repressed in *esr1-1* (Fig 8). Unlike the other repressed genes, *PDF1*.*2* expression in *esr1-1* was reduced at 6 hours but increased above wild-type levels at 24 hours. This suggests, in addition to activation of components of early JA-regulated gene expression, ESR1 may have roles in repression at later stages. We also found repression of JA-regulated genes in *esr1-1* was not due to antagonistic SA-JA crosstalk as SA-regulated marker genes in *esr1-1* showed no increase in their basal or SA induced levels (Fig 7).

The down-regulation of JA-signalling in *esr1-1* likely contributes to its enhanced resistance to *F*. *oxysporum* and increased susceptibility to *A*. *brassicicola* as this pathway confers susceptibility and resistance to these pathogens respectively. For example, the *coi1* and *pft1/med25* mutants, which are highly resistant to *F*. *oxysporum* and susceptible to *A*. *brassicicola*, also exhibit reduced expression of JA-biosynthesis (e.g. *LOX3*, *OPR*), JA-signalling (e.g. *JAZ9*, *JASMONIC ACID CARBOXYL METHYLTRANSFERASE*) and JA-regulated defense/senescence associated genes (e.g. *Thi2*.*1*, *PDF1*.*2*, *CLH1*) under mock, *F*. *oxysporum* or MeJA induced conditions [41, 45, 51]. While defensive components of JA-signalling do contribute positively to *F*. *oxysporum* resistance (e.g. increased *PDF1*.*2*, *Thi2*.*1* expression), global up-regulation of JA-signalling including its non-defensive components promote susceptibility [31, 44, 46, 83]. For example, senescence is proposed to strongly contribute to *F*. *oxysporum* disease symptom development [41]. Unlike the *coi1* and *pft1* mutants which have impairments in growth or development (*coi1* is male sterile with insensitivity to MeJA inhibition of root growth, while *pft1* is delayed in flowering [45, 84]), *esr1-1* is indistinguishable from wild-type plants (Fig 6a–6d and S4 Fig). We also inoculated *esr1-1* with another root-infecting necrotrophic fungal pathogen, *R*. *solani* (isolates AG8 or AG2-1) but found no difference in disease phenotypes compared to wild-type plants (Fig 3a). It is suggested that neither JA or SA signalling pathways contribute to *R*. *solani* resistance or susceptibility in Arabidopsis [42].

Through independent forward genetic screens utilizing abiotic stress inducible promoters, At5g53060 was shown to be nuclear localised and have roles in diverse transcriptional processes including mRNA capping efficiency, polyadenylation site selection, mRNA export, and in the regulation of expression and alternate splicing of some stress inducible genes ([26, 27, 56] this work). Some of these processes, including its nuclear localisation, are mediated through At5g53060 interactions with the RNA PolII CTD interacting phosphatase protein CPL1 [25, 27, 56, 59] whose expression is up-regulated in *esr1-1* (Table 1, S3 Fig). We also tested the expression of *CPL1* and other genes up-regulated in *esr1-1* (*SYTB*, *STP11*, *At3g54160*) for responsiveness to SA or MeJA and found little change (S5 Fig) suggesting they may be regulated at the post transcriptional level by ESR1 or through other signalling pathways.

**Fig 9:**
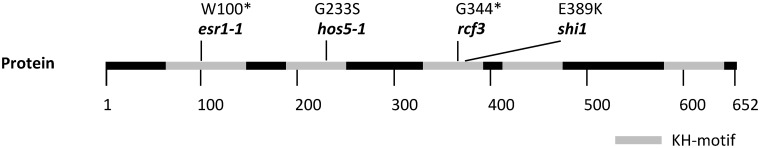
Location and effect of *At5g53060/ESR1/RCF3/SHI1/HOS5* mutations. At5g53060 domain structure with the position and predicted nature of published mutations indicated relative to the first methionine.

We characterised four mutants of At5g53060. The *esr1-1* mutant confers a Tryptophan to STOP codon substitution (W100*) in the first of five At5g53060 KH-domains, *esr1-2* is a null T-DNA insertional inactivation line, while *esr1-3* and *esr1-4* harbour mutations at splice site junctions (Figs 4 and 5). Three other independently identified *At5g53060* alleles confer other mutations. The *rcf3-1* (*regulator of CBF gene expression 1*) mutant isolated through a cold responsive *CBF2* promoter screen confers a Glycine to STOP codon substitution (G344*) within the third At5g53060 KH-domain and displays increased heat tolerance [26]. The *shi1* (*shiny1*) mutant isolated through the salt inducible sulfotransferase *AtSOT12* promoter confers a Glutamic Acid to Lysine substitution (E389K) also within the third KH-domain [27]. The *shi1* mutant is more resistant to ABA during germination and has increased sensitivity to cold stress [27]. Unlike these mutants of At5g53060, the *hos5-1* mutant has increased sensitivity to ABA and salt stress, although tolerance/sensitivity to other abiotic stresses has not yet been tested for this At5g53060 mutant allele [56]. The *hos5-1* (*high osmotic stress gene expression 5*) mutation confers a Glycine to Serine change (G233S) within the second At5g53060 KH-domain. Interestingly, *esr1-1*, *rcf3-1* and *shi1* mutations (Fig 9) disrupt either the first or third KH-domains, both of which along with the fourth domain can interact with CPL1 ([27, 56, 59] this work). Although the *shi1* mutant confers only an amino acid substitution, this change disrupts the CPL1 interaction [27]. The second KH-domain and location of the *hos5-1* mutation (Fig 9) does not interact with CPL1 but may affect RNA binding [56]. This may explain why *hos5-1* is more sensitive to ABA while *shi1* is more tolerant. As with our *esr1-1* and *esr1-2* findings, *rcf3-1* and *shi1* exhibit increased expression of their *Promoter*:*LUC* transgenes but not of their endogenous stress-inducible genes under basal conditions [26, 27]. We did however find *GSTF8* expression was significantly higher than wild-type in *esr1-1* following SA treatment (Fig 2b) suggesting regulation of *GSTF8*:*LUC* promoter and endogenous *GSTF8* differ under basal conditions. Indeed, [27] suggest under basal conditions the ESR1/SHI1-CPL1 complex may associate with other repressors on general transcriptional machinery targeting stress responsive promoters and upon stress inducing conditions this complex is modified.

In summary, we identified roles for the KH-domain RNA-binding protein At5g53060 in JA- and biotic induced stress responses, and define new functions for KH-domain proteins in plants. Further research of interest will be determining At5g53060/ESR1 direct RNA targets, which are yet to be identified, and other proteins it interacts with under specific biotic stresses, in particular those with JA-involvement.

## Supporting Information

S1 Fig Next Generation Mapping locates the *esr1-3* and *esr1-4* loci. (a-b) Whole-genome sequencing of homozygous (a) *esr1-3* or (b) *esr1-4* F_2_s from *esr1* and Ler outcrosses coupled with the Next Generation Mapping tool identifies SNP desserts (underlined region) corresponding to linkage to the *esr1* mutations.
(TIF)

S2 Fig *esr1* mutants have altered thermo-tolerance. (a) *esr1* mutants are more tolerant of heat stress as measured by the proportion of leaf area non-bleached. Seedlings were grown on MS agar plates for 7 days at 21°C, treated at 21°C (control) or 45°C (heat) for 90 minutes, then returned to 21°C for 4 days followed by measurement of bleached area. Values are averages ± SE (n = 10). Asterisks indicate values that are significantly different (**P<0.01 Student’s *t-*test) from wild-type (WT). Similar results were obtained in independent experiments. (b) Average *GSTF8*:*LUC* expression per WT and *esr1-1* seedling per hour after treatment with heat (45°C) or control treatment (21°C). Values are averages ± SE (n = 5) from 7 day old seedlings.
(TIF)

S3 Fig Confirmation of *esr1-1* up-regulated genes by qRT-PCR. Expression confirmation of subset of significantly up-regulated genes in *esr1-1* compared to wild-type (WT) seedlings. Shown are values from 4, 7 and 14 day old seedlings (values are averages ± SE of 3 biological replicates consisting of pools of 20 seedlings, *P*<0.05, all pairs Student’s t-test). Gene expression levels are relative to the internal control *β-actin* genes.
(TIF)

S4 Fig *esr1* mutants do not have altered root sensitivity to JA. Sensitivity of wild-type (WT), *esr1-1* and *esr1-2* seedlings to JA was determined by MeJA inhibition of root growth on control media or media containing 25 uM or 50 uM MeJA. Root elongation of each line when grown on MeJA media was calculated as a percentage relative to their root length on the control. Values are average ± SE for 5 biological replicates consisting of pools of 10 seedlings; *P*<0.05, all pairs Student’s t-test). Similar results were obtained in an independent experiment.
(TIF)

S5 Fig Basal *esr1-1* up-regulated genes are similarly expressed in wild-type and *esr1-1* when treated with MeJA or SA. Fold changes in relative transcript abundance of RNA-seq identified genes in wild-type (WT) and *esr1-1* seedlings 6 and 24 hours post MeJA or SA treatment. Shown are values from 12 day old seedlings (values are averages ± SE of 3 biological replicates consisting of pools of 20–30 seedlings). Transcript levels of each gene of interest following MeJA or SA treatment were normalised against the internal control *β-actin* genes and expressed relative to the normalised levels in mock-treated WT or *esr1-1* seedlings. The numbers on each bar show fold increase or fold decrease caused by each treatment relative to mock-treated plants. *PDF1*.*2* and *PR1* were used as marker genes for MeJA and SA treatment respectively.
(TIF)

S1 Table Primers used for construct generation, mapping and qRT-PCR. (XLSX)

S2 Table esr1-1 significantly down-regulated genes. (XLSX)

S3 Table esr1-1 significantly up-regulated genes. (XLSX)

S4 Table Gene Ontology (GO) terms significantly over-represented in genes down-regulated in *esr1-1* versus wild-type. (XLSX)

S5 Table Gene Ontology (GO) terms significantly over-represented in genes up-regulated in *esr1-1* versus wild-type. (XLSX)
