# Supplementary figures and images for: The Arabidopsis KH-Domain RNA-Binding Protein ESR1 Functions in Components of Jasmonate Signalling, Unlinking Growth Restraint and Resistance to Stress
Source: PLoS One. 2015 May 18;10(5):e0126978. doi: 10.1371/journal.pone.0126978 (PMC4436139; doi:10.1371/journal.pone.0126978)

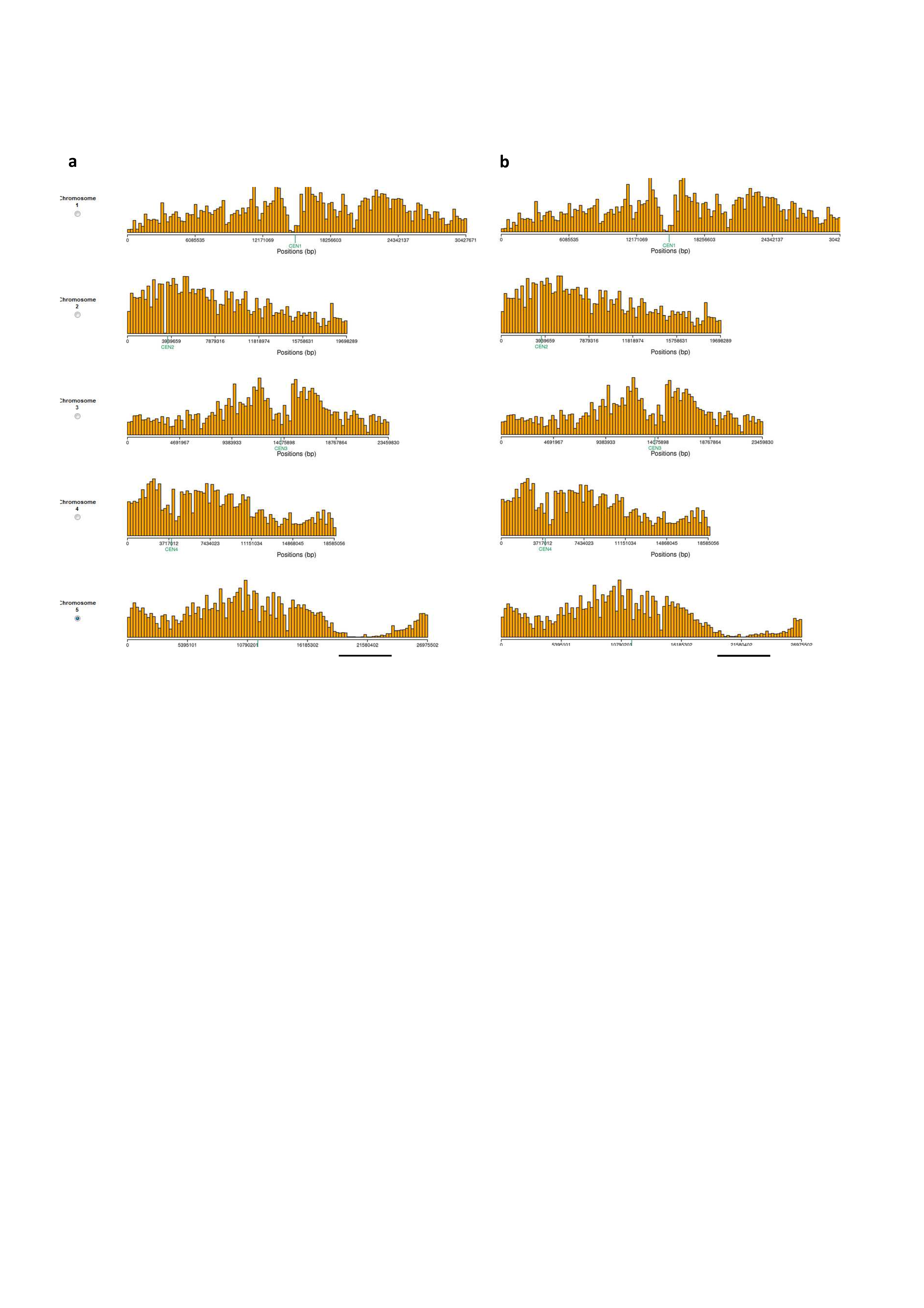

Supplement: S1 Fig — (a-b) Whole-genome sequencing of homozygous (a) esr1-3 or (b) esr1-4 F2s from esr1 and Ler outcrosses coupled with the Next Generation Mapping tool identifies SNP desserts (underlined region) corresponding to linkage to the esr1 mutations. (TIF) [file pone.0126978.s001.tif]

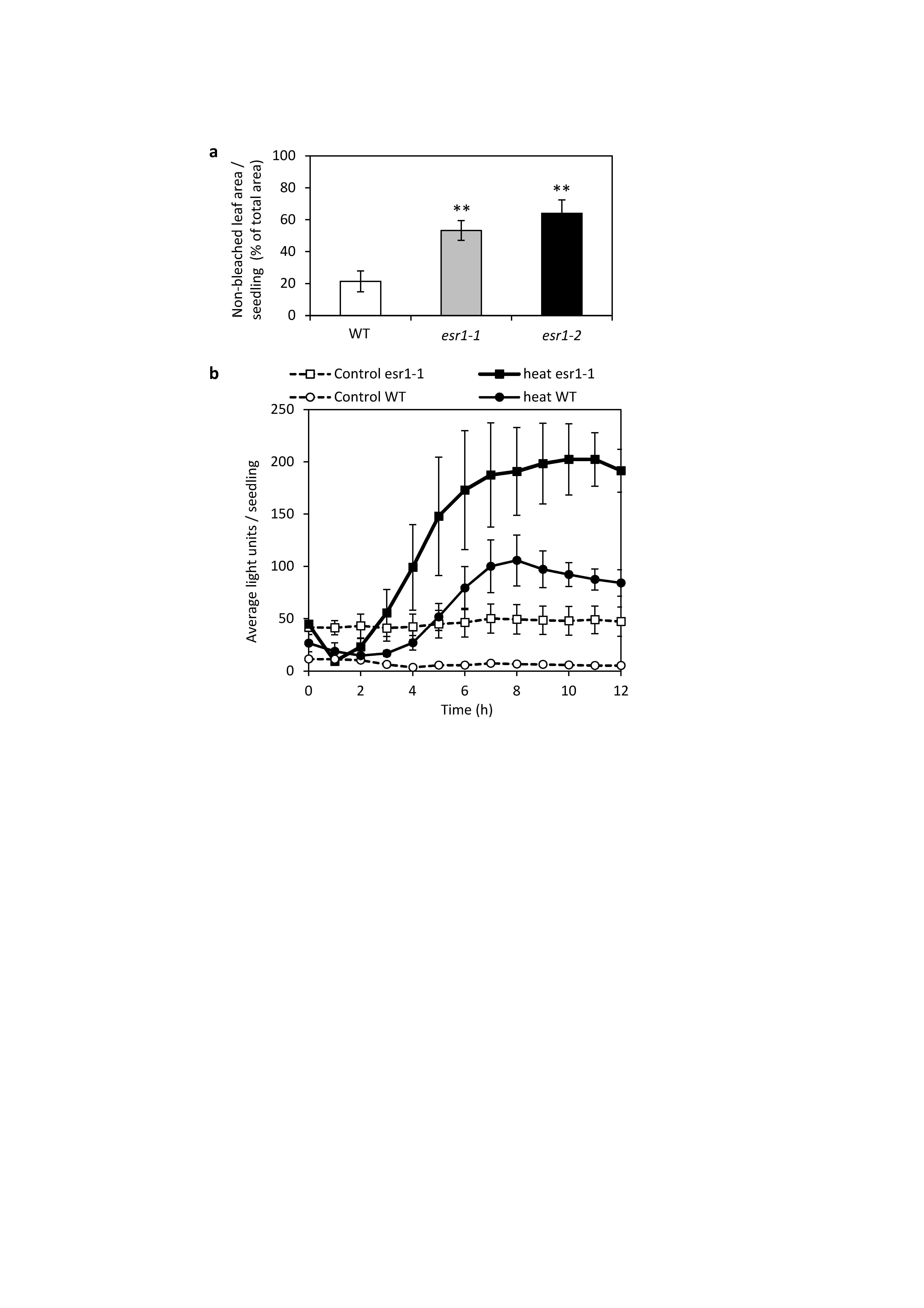

Supplement: S2 Fig — (a) esr1 mutants are more tolerant of heat stress as measured by the proportion of leaf area non-bleached. Seedlings were grown on MS agar plates for 7 days at 21°C, treated at 21°C (control) or 45°C (heat) for 90 minutes, then returned to 21°C for 4 days followed by measurement of bleached area. Values are averages ± SE (n = 10). Asterisks indicate values that are significantly different (**P<0.01 Student’s t-test) from wild-type (WT). Similar results were obtained in independent experiments. (b) Average GSTF8:LUC expression per WT and esr1-1 seedling per hour after treatment with heat (45°C) or control treatment (21°C). Values are averages ± SE (n = 5) from 7 day old seedlings. (TIF) [file pone.0126978.s002.tif]

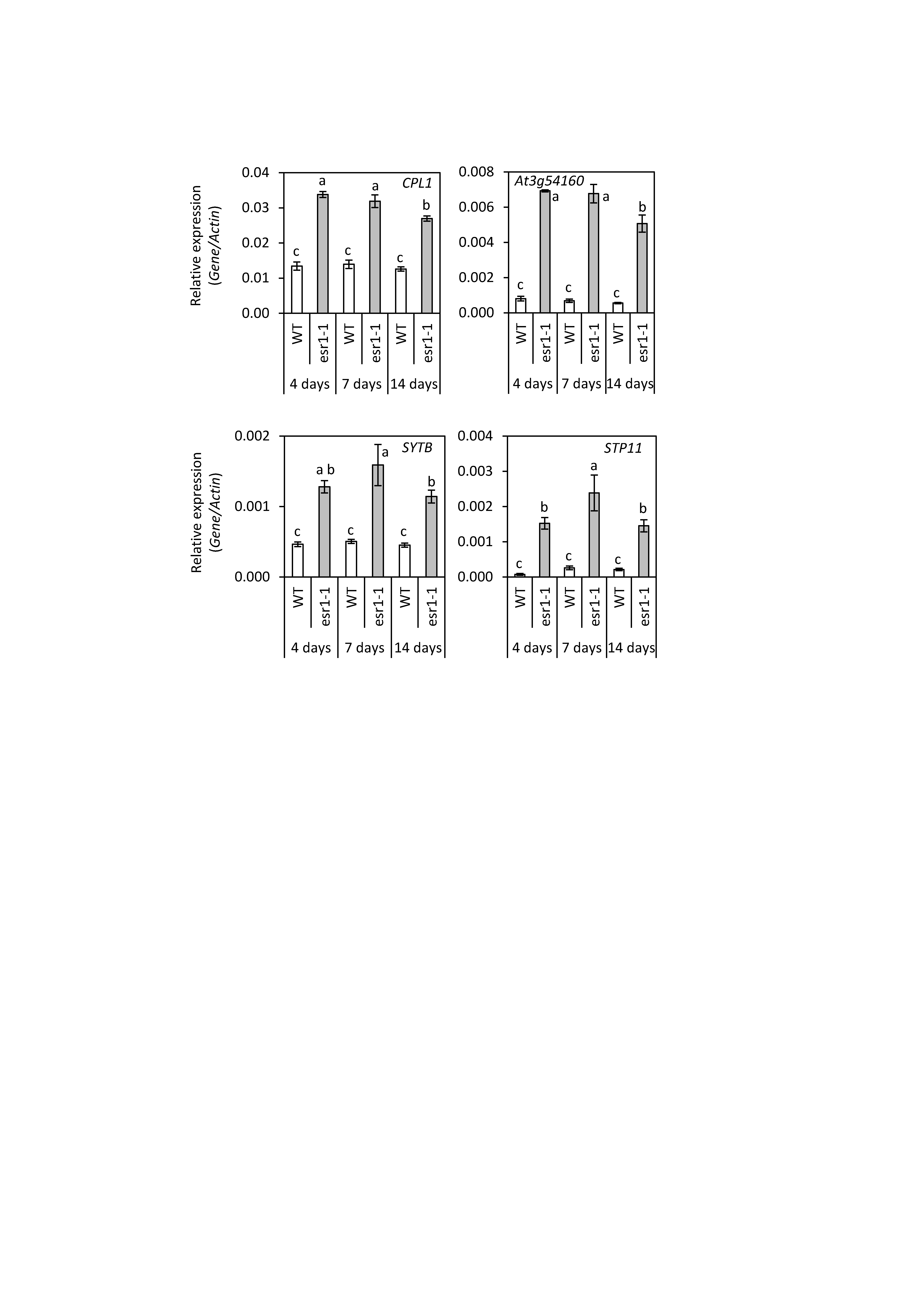

Supplement: S3 Fig — Expression confirmation of subset of significantly up-regulated genes in esr1-1 compared to wild-type (WT) seedlings. Shown are values from 4, 7 and 14 day old seedlings (values are averages ± SE of 3 biological replicates consisting of pools of 20 seedlings, P<0.05, all pairs Student’s t-test). Gene expression levels are relative to the internal control β-actin genes. (TIF) [file pone.0126978.s003.tif]

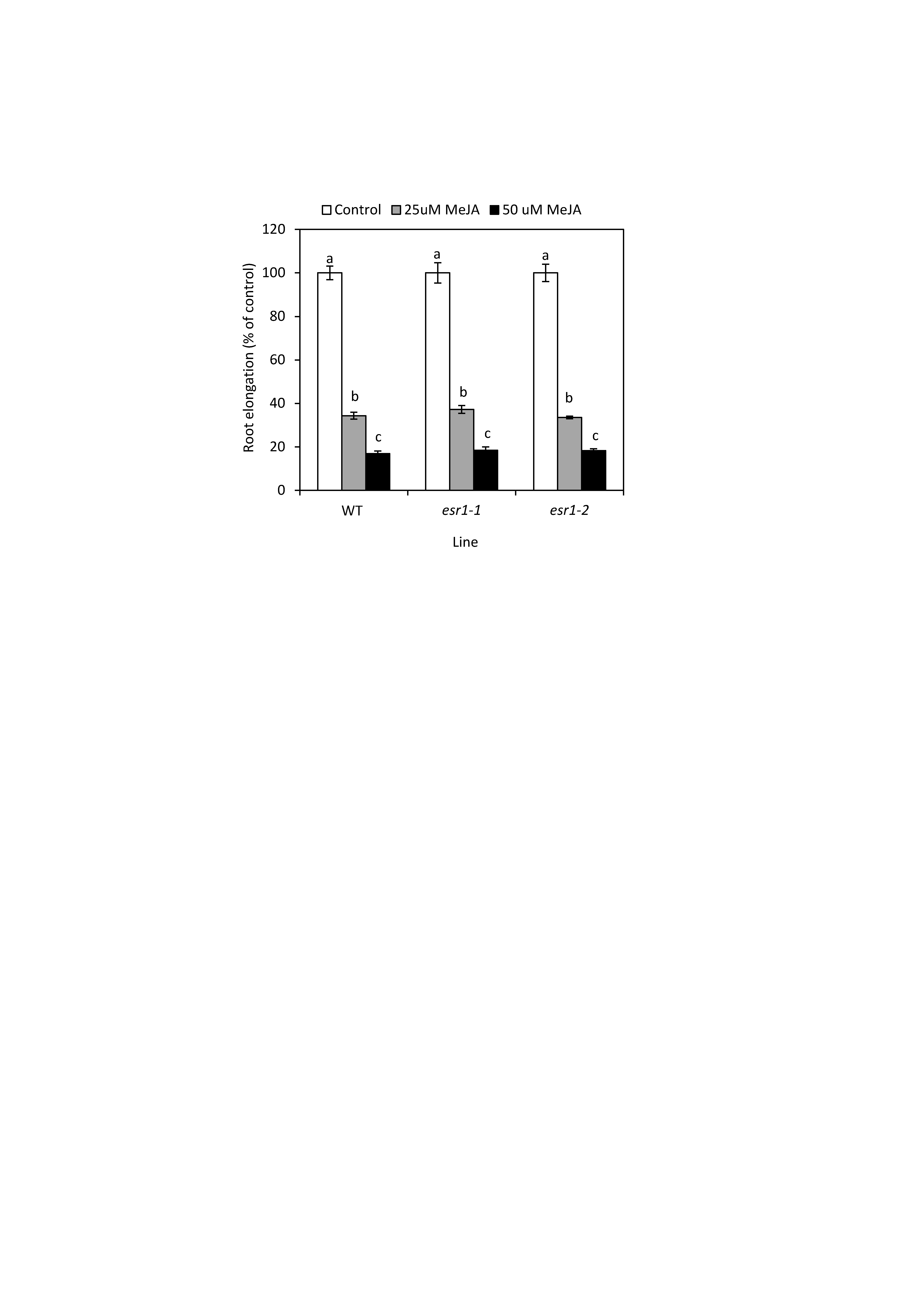

Supplement: S4 Fig — Sensitivity of wild-type (WT), esr1-1 and esr1-2 seedlings to JA was determined by MeJA inhibition of root growth on control media or media containing 25 uM or 50 uM MeJA. Root elongation of each line when grown on MeJA media was calculated as a percentage relative to their root length on the control. Values are average ± SE for 5 biological replicates consisting of pools of 10 seedlings; P<0.05, all pairs Student’s t-test). Similar results were obtained in an independent experiment. (TIF) [file pone.0126978.s004.tif]

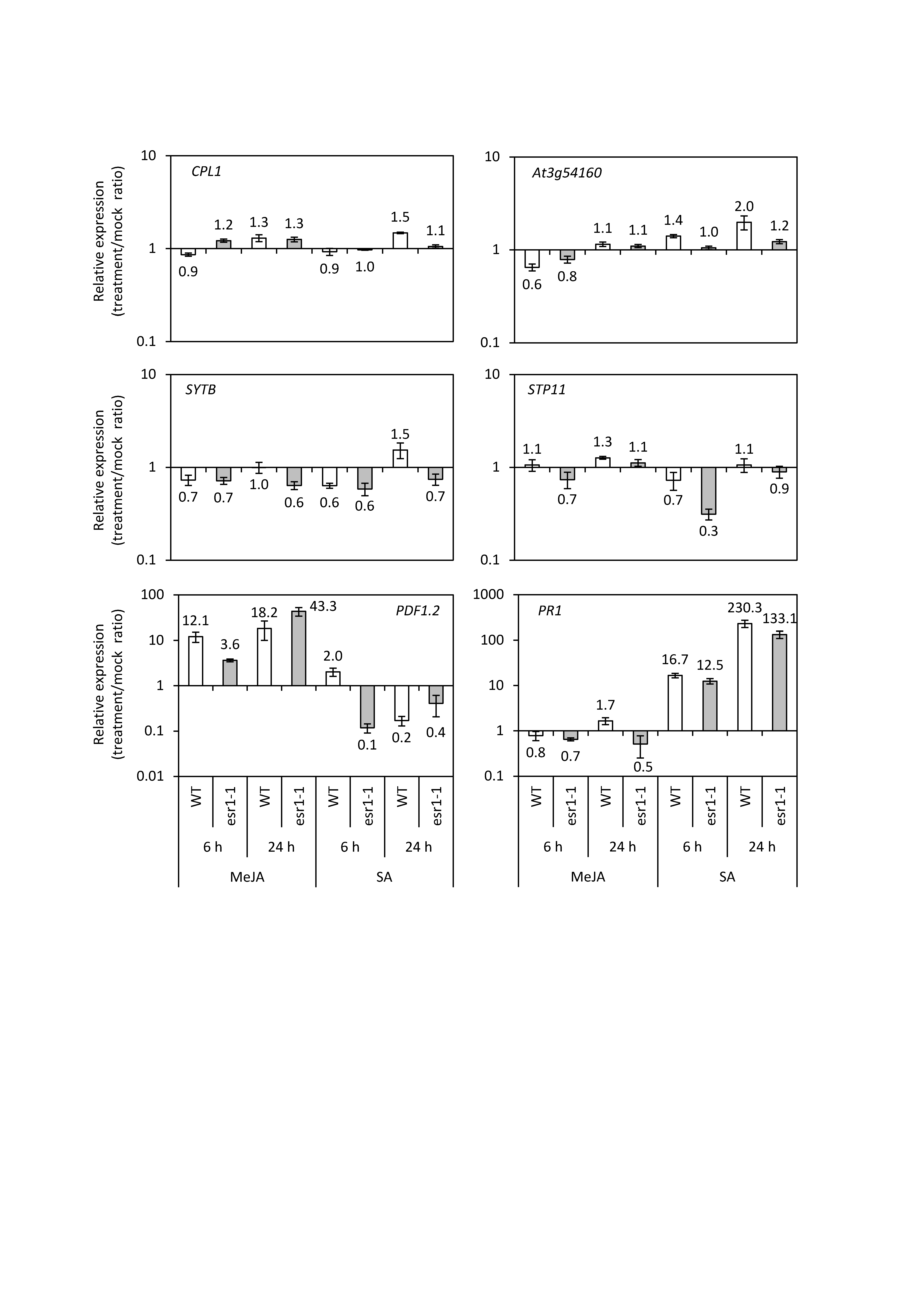

Supplement: S5 Fig — Fold changes in relative transcript abundance of RNA-seq identified genes in wild-type (WT) and esr1-1 seedlings 6 and 24 hours post MeJA or SA treatment. Shown are values from 12 day old seedlings (values are averages ± SE of 3 biological replicates consisting of pools of 20–30 seedlings). Transcript levels of each gene of interest following MeJA or SA treatment were normalised against the internal control β-actin genes and expressed relative to the normalised levels in mock-treated WT or esr1-1 seedlings. The numbers on each bar show fold increase or fold decrease caused by each treatment relative to mock-treated plants. PDF1.2 and PR1 were used as marker genes for MeJA and SA treatment respectively. (TIF) [file pone.0126978.s005.tif]
